# Clinical outcomes of endovascular treatment for acute basilar artery occlusion patients with extremely severe symptoms

**DOI:** 10.3389/fneur.2025.1736679

**Published:** 2026-01-07

**Authors:** Yongtao Guo, Yuqian Xie, Miao Chai, Linyu Li, Shuangzhi Wang, Sheng Zhou, Haoxuan Zhu, Gaoming Li, Lilan Wang, Chunye Chen, Mingyang Chen, Yuhan Fan, Qiuyi Yang, Yihui Yang, Yian Chen, Binghan Wang, Guanting Heng, Xuexiao Zhao, Chawen Ding, Jiaxing Song, Jie Tang, Zhenqian Liu

**Affiliations:** 1 Department of Neurology, The Affiliated Huaian No.1 People's Hospital of Nanjing Medical University, Nanjing Medical University, Huai'an, Jiangsu, China; 2 Department of Neurology, Huai‘an First People's Hospital, Huai'an, China; 3 Department of Neurosurgery, The Second Affiliated Hospital of Guangxi Medical University, Nanning, China; 4 Department of Neurology, Lanzhou University Second Hospital, Lanzhou, China; 5 Department of Neurology, Xinqiao Hospital and The Second Affiliated Hospital, Army Medical University (Third Military Medical University), Chongqing, China; 6 Department of Neurology, The Second Affiliated Hospital of Chongqing Medical University, Chongqing, China; 7 Department of Neurology, The Third Hospital of Mianyang, Mianyang, China; 8 Department of Neurology, Renhuai People's Hospital, Renhuai, China; 9 Department of Neurology, The Affiliated Yongchuan Hospital of Chongqing Medical University, Chongqing, China; 10 Department of Neurology, The 908th Hospital of Chinese People's Liberation Army Joint Logistic Support Force, Nanchang, China; 11 Department of Neurology, The People's Hospital of Jianyang City, Jianyang, China; 12 Department of Neurology, The Second Affiliated Hospital of Xuzhou Medical University, Xuzhou, China

**Keywords:** endovascular, treatment, stroke, stroke severity (NIHSS), prognosis, large vessel occlusion

## Abstract

**Objective:**

This study aimed to investigate the effectiveness and safety of endovascular treatment (EVT) in acute basilar artery occlusion (ABAO) patients with extremely severe symptoms [National Institutes of Health Stroke Scale (NIHSS) score >25] in the real world.

**Methods:**

This study was a subgroup analysis of a prospective multicenter cohort study (EVT for Acute Basilar Artery Occlusion Study, BASILAR registry). Patients were categorized into EVT and standard medical treatment (SMT) groups. The primary effectiveness outcome was the distribution of modified Rankin Scale (mRS) scores at 90 days. Safety outcomes included 90-day mortality and symptomatic intracerebral hemorrhage (sICH) within 48 h.

**Results:**

Among 436 ABAO patients with extremely severe symptoms, 342 (78.4%) underwent EVT. Compared with SMT, EVT was significantly associated with greater odds of favorable shift in mRS distribution [adjusted OR (aOR) 3.98, 95% CI 2.02–7.84, *P* < 0.001] and lower mortality (aOR 0.26, 95% CI 0.13–0.53, *P* < 0.001). All outcomes remained consistent after propensity score matching (PSM). No significant difference in sICH was observed between groups after PSM. Furthermore, shorter onset to treatment time and puncture to reperfusion time were associated with higher predicted probabilities of achieving mRS 0–3 and lower predicted probabilities of mortality. Additionally, the effectiveness and safety of EVT decreased progressively with increasing baseline stroke severity.

**Conclusions:**

In ABAO patients with extremely severe symptoms, EVT demonstrated superior functional outcomes and lower mortality. Minimizing onset to treatment time and puncture to reperfusion time is essential for optimizing clinical outcomes in this patient population.

## Introduction

Despite accounting for only 1% of all ischemic strokes and 5–10% of large vessel occlusions (LVO), acute basilar artery occlusion (ABAO) is associated with exceptionally high rates of disability and mortality (1, 2). The advent of endovascular treatment (EVT) has provided a promising therapeutic approach for patients with ABAO. Four landmark randomized controlled trials (RCTs) evaluated the efficacy and safety of EVT compared with standard medical treatment (SMT) in patients with ABAO. Although the BASICS (3) and BEST (4) trials failed to demonstrate the superiority of EVT over SMT, these pivotal studies laid the foundation for subsequent research. More recently, the ATTENTION (5) and BAOCHE (6) trials have demonstrated compelling evidence supporting the clinical efficacy of EVT in ABAO patients. Consistent with these findings, our prospective cohort study [Endovascular Treatment for Acute Basilar Artery Occlusion Study (BASILAR)] (7) revealed that EVT was significantly associated with improved functional outcomes and decreased mortality in patients with ABAO.

Previous studies found that EVT seemed to be safer and more effective for ABAO patients with mild to moderate symptoms compared to those with more severe symptoms (8, 9). These findings suggest that clinical severity at admission is associated with functional outcomes after EVT. Notably, while a prior study indicated that EVT was associated with improved functional outcomes even in ABAO patients with severe symptoms [National Institutes of Health Stroke Scale (NIHSS) score > 20] (10), dedicated studies investigating the effectiveness of EVT in ABAO patients with extremely severe symptoms (NIHSS score > 25) remain limited. This patient population is frequently excluded from clinical trials or analyzed only as subgroups. Consequently, whether EVT confers clinical benefit in ABAO patients with extremely severe symptoms remains unknown.

Therefore, this study aimed to investigate the effectiveness and safety of EVT in ABAO patients with extremely severe symptoms in the real world.

## Methods

### Study design and participants

The data analyzed in this study were obtained from the BASILAR registry (7). The BASILAR registry was a prospective multicenter cohort study that enrolled patients with ABAO from 47 stroke centers across China between January 2014 and May 2019. This study followed the ethical principles of the Helsinki Declaration and was approved by the ethics committees of all participating centers. All patients or their legal representatives provided written informed consent before enrollment. The inclusion criteria of enrolled centers have been published in the BASILAR main study.

The inclusion criteria for this study were as follows: (1) age ≥ 18 years; (2) ABAO confirmed by computed tomographic angiography (CTA), magnetic resonance angiography (MRA), or digital subtraction angiography (DSA); (3) time from symptom onset or last known well within 24 h; (4) ability to provide informed consent. The exclusion criteria were: (1) pre-stroke modified Rankin scale (mRS) score > 2; (2) neuroimaging evidence of intracranial hemorrhage on initial presentation; (3) absence of 90-day follow-up data; (4) current pregnancy or lactation; (5) severe, advanced or terminal illnesses.

### Data collection

The following data were collected for all patients: demographics characteristics, medical history, prodromal symptoms, systolic blood pressure (SBP), stroke severity at admission, pre-stroke modified Rankin Scale score (mRS) score, neuroimaging findings, stroke etiology, intravenous thrombolysis (IVT) administration, workflow (time from symptom onset to imaging and treatment), EVT characteristics, anesthesia type, reperfusion status, and functional outcomes at 90 days and 1 year. Stroke severity of ABAO was dichotomized into extremely severe symptoms (i.e., NIHSS score > 25), moderate to severe symptoms (ie, NIHSS score 10–25) and mild to moderate symptoms (i.e., NIHSS score <10) groups using baseline NIHSS score from the BASILAR study. Stroke etiology was classified according to the Trial of ORG 10172 in Acute Stroke Treatment (TOAST) classification system. Collateral circulation was evaluated using the American Society of Interventional and Therapeutic Neuroradiology/Society of Interventional Radiology (ASITN/SIR) grading system, with collateral status categorized as poor (grades 0–1), moderate (grade 2), or good (grades 3–4). The extent of ischemic injury was assessed using the posterior circulation-Alberta Stroke Program Early Computed Tomography Score (pc-ASPECTS). Reperfusion status was evaluated on the final angiogram using the modified Treatment in Cerebral Ischemia (mTICI) score, with successful reperfusion defined as mTICI 2b-3.

### Outcomes

The primary effectiveness outcome was the distribution of mRS scores at 90 days (ranging from 0 [no symptoms] to 6 [death]). Secondary effectiveness outcomes included the proportion of patients achieving mRS 0–3, mRS 0–2, and mRS 0–1 at 90 days. Safety outcomes included 90-day mortality and symptomatic intracerebral hemorrhage (sICH) within 48 h confirmed by computed tomography (CT) or magnetic resonance imaging (MRI). sICH was defined according to the Heidelberg Bleeding Classification (11).

### Statistical analysis

Normality of continuous variables was assessed using the Kolmogorov-Smirnov test. Normally distributed continuous variables were presented as mean ± standard deviation (SD), while non-normally distributed variables were presented as median [interquartile range (IQR)]. Categorical variables were expressed as frequencies and percentages. Between-group comparisons were performed using Student's *t*-test for normally distributed continuous variables or Mann–Whitney U test for non-normally distributed variables. Categorical variables were compared using the chi-square test or Fisher's exact test when appropriate.

Logistic regression analyses were performed to assess the association between EVT and clinical outcomes. The shift in mRS score distribution was analyzed using ordinal logistic regression. Binary logistic regression was performed for dichotomous outcomes including mRS 0–3, mRS 0–2, mRS 0–1, sICH, and mortality. Results were reported as odds ratios (OR) with 95% confidence intervals (CIs). Multivariable regression analyses were performed adjusting for covariates selected based on clinical relevance and baseline characteristics. For analyses of patients with extremely severe symptoms (NIHSS score > 25) and moderate to severe symptoms (NIHSS score 10–25), covariates included age, sex, systolic blood pressure (SBP), baseline NIHSS, baseline pc-ASPECTS, smoking history, ASITN/SIR grade, stroke etiology, occlusion site, and IVT. For analyses of patients receiving EVT, we adjusted for age, sex, baseline pc-ASPECTS, atrial fibrillation (AF), coronary heart disease (CHD), ASITN/SIR grade, stroke etiology, anesthesia type, and reperfusion status.

Two separate propensity score matching (PSM) analyses were conducted to minimize selection bias and balance baseline characteristics between treatment groups. For patients with extremely severe symptoms, propensity scores were estimated using multivariable logistic regression with age, SBP, smoking history, baseline pc-ASPECTS, occlusion site, and IVT as covariates. For patients with moderate to severe symptoms, propensity scores incorporated SBP, baseline NIHSS, baseline pc-ASPECTS, and occlusion site as covariates. For both analyses, 1:2 nearest neighbor matching algorithm without replacement was employed to compare outcomes between SMT and EVT groups. Matching was conducted using a caliper width of 0.2 standard deviations of the logit of the propensity score. Detailed information regarding both PSM analyses was provided in Methods S1 and S2, respectively.

Marginal effects plots were constructed to visualize the association between key variables (onset to treatment time and puncture to reperfusion time) and clinical outcomes (mRS 0–3 and mortality), adjusting for the same covariates used in the multivariable logistic regression models. Subgroup analyses were performed to assess the effects of EVT on mRS distribution in specific subgroups. Interaction terms between treatment groups and subgroup indicators were incorporated into the models to evaluate treatment effect heterogeneity across subgroups. Given the minimal missing data for key variables, no imputation methods were employed. A two-tailed *P*-value <0.05 was considered statistically significant. Statistical analyses were conducted using IBM SPSS Statistics version 27.0 (IBM Corp., Armonk, NY, USA) and R version 4.4.1 (R Foundation for Statistical Computing, Vienna, Austria).

## Results

### Baseline characteristics of patients with extremely severe symptoms (NIHSS score > 25)

Among 829 patients with ABAO, 436 patients with extremely severe symptoms were included in the analysis (Figure S1). Of these, 342 (78.4%) underwent EVT. The median age was 65 (IQR, 57–74) years, and 317 (72.7%) patients were male. Compared with the SMT group, patients treated with EVT had higher proportions of AF (25.7% vs. 16.0%, *P* = 0.048) and smoking history (35.7% vs. 14.9%, *P* < 0.001), lower SBP (150 vs. 160 mmHg, *P* < 0.001), and higher baseline pc-ASPECTS (8 vs. 7, *P* = 0.003). EVT patients were less frequently to receive IVT (19.3% vs. 34.0%, *P* = 0.002). Additionally, significant differences were observed between groups in stroke etiology (*P* = 0.003) and occlusion sites (*P* = 0.002). No significant differences were observed in other baseline characteristics between treatment groups. Among EVT patients, the median onset to puncture time was 320 (IQR, 220–487) min, and the median puncture to reperfusion time was 102 (IQR, 70–142) min. General anesthesia was administered in 152 (45.0%) patients, and successful reperfusion was achieved in 267 (78.1%) patients. After PSM, baseline characteristics were well-balanced between the two groups. Other details were presented in Table 1.

**Table 1:** Comparison of baseline characteristics between SMT and EVT groups in ABAO patients with extremely severe symptoms (NIHSS score > 25).

**Characteristics**	**Before PSM**				**After PSM (1:2)**			
	**All patients (*****n*** = **436)**	**SMT (*****n*** = **94)**	**EVT (*****n*** = **342)**	***P-*** **value**	**All patients (*****n*** = **255)**	**SMT (*****n*** = **91)**	**EVT (*****n*** = **164)**	***P-*** **value**
Age, *y*, median (IQR)	65 (57–74)	68 (59–76)	65 (57–74)	0.126	66 (59–74)	66 (59–75)	66 (58–74)	0.730
Sex, male, *n* (%)	317 (72.7)	64 (68.1)	253 (74.0)	0.256	172 (67.5)	62 (68.1)	110 (67.1)	0.863
**Medical history**, ***n*** **(%)**								
Hypertension	303 (69.5)	65 (69.1)	238 (69.6)	0.934	184 (72.2)	64 (70.3)	120 (73.2)	0.628
Diabetes mellitus	99 (22.7)	20 (21.3)	79 (23.1)	0.709	54 (21.2)	19 (20.9)	35 (21.3)	0.931
Hyperlipidemia	134 (30.7)	30 (31.9)	104 (30.4)	0.779	79 (31.0)	30 (33.0)	49 (29.9)	0.609
Smoking	136 (31.2)	14 (14.9)	122 (35.7)	<0.001	41 (16.1)	14 (15.4)	27 (16.5)	0.822
Ischemic stroke	102 (23.4)	25 (26.6)	77 (22.5)	0.408	57 (22.4)	23 (25.3)	34 (20.7)	0.404
AF	103 (23.6)	15 (16.0)	88 (25.7)	0.048	58 (22.7)	15 (16.5)	43 (26.2)	0.076
CHD	81 (18.6)	12 (12.8)	69 (20.2)	0.102	37 (14.5)	12 (13.2)	25 (15.2)	0.655
Prodromal transient ischemic stroke or minor stroke	194 (44.5)	45 (47.9)	149 (43.6)	0.457	116 (45.5)	45 (49.5)	71 (43.3)	0.344
SBP, mmHg, median (IQR)^a^	151 (135–168)	160 (145–172)	150 (132–166)	<0.001	156 (140–171)	160 (144–172)	154 (140–170)	0.319
Baseline NIHSS score, median (IQR)	33 (30–35)	32 (30–35)	33 (30–35)	0.504	32 (30–35)	32 (30–35)	33 (30–35)	0.807
Baseline pc-ASPECTS, median (IQR)^b^	8 (6–9)	7 (6–8)	8 (6–9)	0.003	7 (6–8)	7 (6–8)	7 (6–8)	0.969
**ASITN/SIR grade**, ***n*** **(%)**								
0–1	332 (76.1)	72 (76.6)	260 (76.0)	0.170	199 (78.0)	69 (75.8)	130 (79.3)	0.131
2	82 (18.8)	14 (14.9)	68 (19.9)		43 (16.9)	14 (15.4)	29 (17.7)	
3–4	22 (5.0)	8 (8.5)	14 (4.1)		13 (5.1)	8 (8.8)	5 (3.0)	
**Pre-stroke mRS score**								
0	369 (84.6)	81 (86.2)	288 (84.2)	0.757	223 (87.5)	79 (86.8)	144 (87.8)	0.958
1	41 (9.4)	7 (7.4)	34 (9.9)		18 (7.1)	7 (7.7)	11 (6.7)	
2	26 (6.0)	6 (6.4)	20 (5.8)		14 (5.5)	5 (5.5)	9 (5.5)	
**Stroke etiology**, ***n*** **(%)**								
LAA	261 (59.9)	55 (58.5)	206 (60.2)	0.003	146 (57.3)	54 (59.3)	92 (56.1)	0.063
CE	123 (28.2)	19 (20.2)	104 (30.4)		72 (28.2)	19 (20.9)	53 (32.3)	
Others	52 (11.9)	20 (21.3)	32 (9.4)		37 (14.5)	18 (19.8)	19 (11.6)	
**Occlusion site**, ***n*** **(%)**								
Distal basilar artery	162 (37.2)	31 (33.0)	131 (38.3)	0.002	100 (39.2)	29 (31.9)	71 (43.3)	0.002
Middle basilar artery	142 (32.6)	45 (47.9)	97 (28.4)		88 (34.5)	45 (49.5)	43 (26.2)	
Proximal basilar artery	56 (12.8)	6 (6.4)	50 (14.6)		28 (11.0)	6 (6.6)	22 (13.4)	
Vertebral artery-V4 segment	76 (17.4)	12 (12.8)	64 (18.7)		39 (15.3)	11 (12.1)	28 (17.1)	
IVT, *n* (%)	98 (22.5)	32 (34.0)	66 (19.3)	0.002	80 (31.4)	30 (33.0)	50 (30.5)	0.683
Onset to imaging time, min, median (IQR)	210 (100–354)	199 (94–370)	214 (102–352)	0.900	219 (100–356)	195 (94–360)	220 (100–356)	0.762
Onset to treatment time, min, median (IQR)	247 (140–394)	240 (134–408)	248 (141–394)	0.982	247 (135–394)	236 (132–394)	251 (142–400)	0.738
Onset to puncture time, min, median (IQR)	NA	NA	320 (220–487)	NA	NA	NA	333 (229–495)	NA
Puncture to reperfusion time, min, median (IQR)	NA	NA	102 (70–142)	NA	NA	NA	101 (76–150)	NA
General anesthesia, *n* (%)^c^	NA	NA	152 (45.0)	NA	NA	NA	92 (57.1)	NA
mTICI score of 2b-3 at final angiogram, *n* (%)	274 (62.8)	7 (7.4)	267 (78.1)	<0.001	136 (53.3)	7 (7.7)	129 (78.7)	<0.001

^a^Data were missing for 2 patients in the EVT group.

^b^Data were missing for 2 patients in the SMT group and 4 patients in the EVT group.

^c^Data were missing for 4 patients in the EVT group.

AF, atrial fibrillation; ASITN/SIR, American Society of Interventional and Therapeutic Neuroradiology/Society of Interventional Radiology; CE, cardioembolism; CHD, coronary heart disease; CI, confidence interval; EVT, endovascular treatment; IQR, interquartile range; IVT, intravenous thrombolysis; LAA, large artery atherosclerosis; mRS, modified Rankin Scale; mTICI, modified Treatment in Cerebral Infarction; NA, not applicable; NIHSS, National Institutes of Health Stroke Scale; pc-ASPECTS, posterior circulation-Alberta Stroke Program Early Computed Tomography Score; PSM, propensity score matching; SBP, systolic blood pressure; SMT, standard medical treatment.

### Clinical outcomes of patients with extremely severe symptoms (NIHSS score > 25; SMT group vs. EVT group)

**Figure 1:**
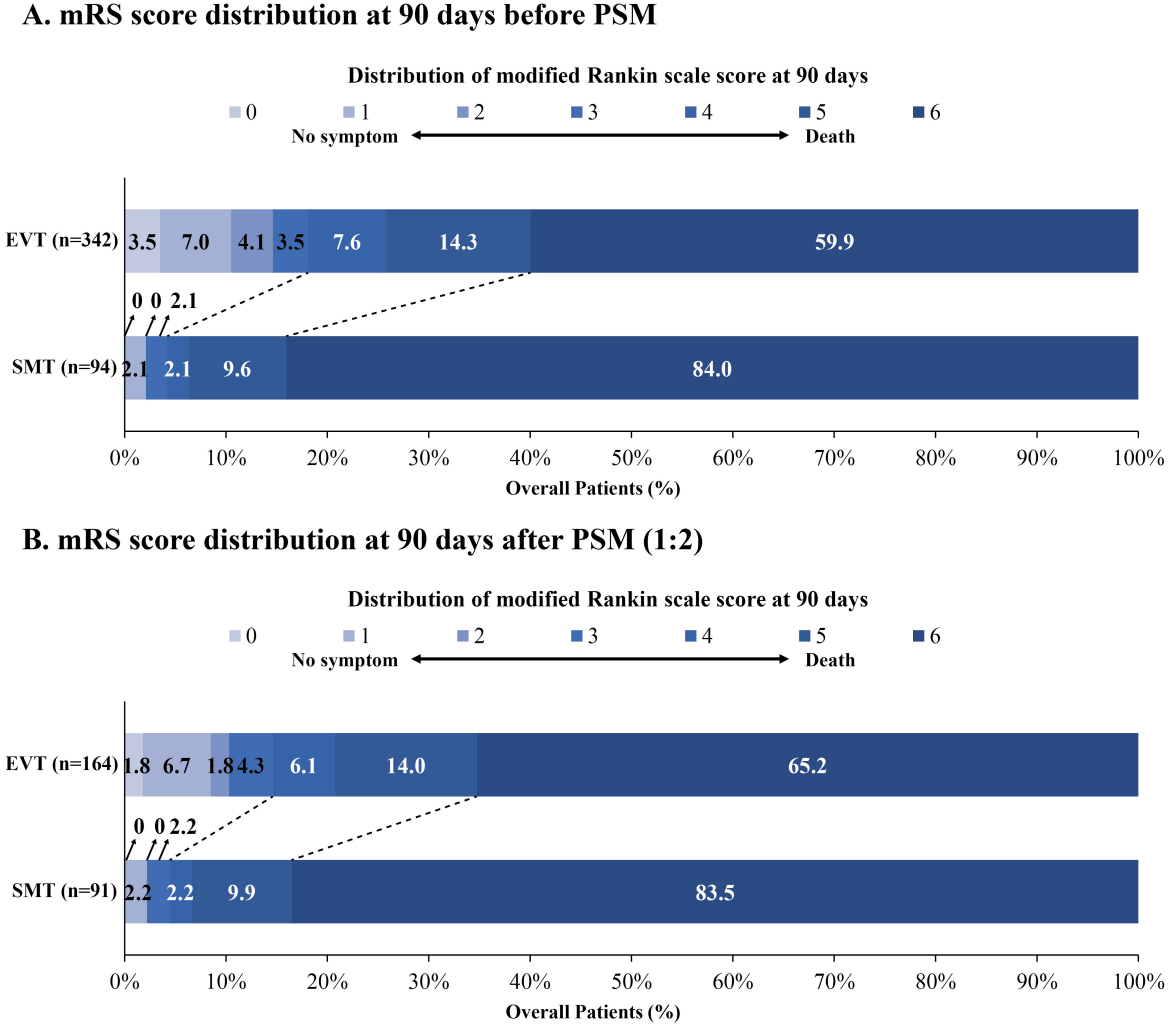
Distribution of modified Rankin scale score at 90 days in ABAO patients with extremely severe symptoms (NIHSS score >25). The distributions of mRS scores at 90 days in ABAO patients with extremely severe symptoms are presented for the SMT and EVT groups before **(A)** and after **(B)** PSM. Abbreviations: ABAO, acute basilar artery occlusion; EVT, endovascular treatment; mRS, modified Rankin scale; PSM, propensity score matching; SMT, standard medical treatment.

The median mRS score was 6 (IQR, 6-6) in the SMT group and 6 (IQR, 4–6) in the EVT group. Patients receiving EVT demonstrated significantly greater odds of favorable shift in mRS distribution compared to those receiving SMT [adjusted OR (aOR) 3.98, 95% CI 2.02–7.84, *P* < 0.001; Figure 1A and Table 2]. Compared with the SMT group, the EVT group showed significantly higher odds of achieving mRS 0–3 (aOR 4.18, 95% CI 1.28–13.67, *P* = 0.018) and mRS 0–2 (aOR 6.58, 95% CI 1.39–31.22, *P* = 0.018). The incidence of sICH within 48 hours was higher in the EVT group (aOR 8.80, 95% CI 1.14–67.70, *P* = 0.037). However, mortality was significantly lower in the EVT group than in the SMT group (aOR 0.26, 95% CI 0.13–0.53, *P* < 0.001). Following PSM, this difference in sICH was no longer statistically significant. Other results remained consistent before and after PSM (Figure 1B and Table 2). Long-term follow-up at 1 year demonstrated comparable clinical outcomes between the two groups (Figure S2 and Table S1). Additionally, we compared the effectiveness and safety of EVT vs. SMT among patients with NIHSS score of 10–25, with detailed results presented in Tables S2–S4.

**Table 2:** Clinical outcomes at 90 days between SMT and EVT groups in ABAO patients with extremely severe symptoms (NIHSS score > 25).

**Clinical outcomes**	**Before PSM**				**After PSM (1:2)**			
	**SMT (*****n*** = **94)**, ***n*** **(%)**	**EVT (*****n*** = **342)**, ***n*** **(%)**	**Adjusted OR (95% CI)** ^b^	***P*** **-value**	**SMT (*****n*** = **91)**, ***n*** **(%)**	**EVT (*****n*** = **164)**, ***n*** **(%)**	**Adjusted OR (95% CI)** ^b^	***P*** **-value**
mRS score at 90d^c^, median (IQR)	6 (6–6)	6 (4–6)	3.98 (2.02–7.84)	<0.001	6 (6–6)	6 (5–6)	3.53 (1.73–7.20)	<0.001
mRS 0–3 at 90d^d^	4 (4.3)	62 (18.1)	4.18 (1.28–13.67)	0.018	4 (4.4)	24 (14.6)	4.41 (1.27–15.29)	0.019
mRS 0–2 at 90d^d^	2 (2.1)	50 (14.6)	6.58 (1.39–31.22)	0.018	2 (2.2)	17 (10.4)	7.24 (1.39–37.80)	0.019
mRS 0–1 at 90d^d^	2 (2.1)	36 (10.5)	4.36 (0.90–21.13)	0.067	2 (2.2)	14 (8.5)	5.50 (1.01–29.85)	0.048
Mortality at 90d^d^	79 (84.0)	205 (59.9)	0.26 (0.13–0.53)	<0.001	76 (83.5)	107 (65.2)	0.30 (0.14–0.64)	0.002
sICH within 48h^a, d^	1 (1.1)	35 (10.5)	8.80 (1.14–67.70)	0.037	1 (1.1)	13 (8.1)	8.09 (0.97–67.23)	0.053

^a^Data were missing for 10 patients in the EVT group.

^b^adjusted for age, sex, SBP, baseline NIHSS, baseline pc-ASPECTS, smoking history, ASITN/SIR grade, stroke etiology, occlusion site, and IVT.

^c^The common odds ratio was estimated from an ordinal logistic regression model.

^d^The odds ratios were estimated from a binary logistic regression model.

ASITN/SIR, American Society of Interventional and Therapeutic Neuroradiology/Society of Interventional Radiology; CI, confidence interval; EVT, endovascular treatment; IQR, interquartile range; IVT, intravenous thrombolysis; mRS, modified Rankin Scale; NIHSS, National Institutes of Health Stroke Scale; OR, odds ratio; pc-ASPECTS, posterior circulation-Alberta Stroke Program Early CT Score; PSM, propensity score matching; SBP, systolic blood pressure; sICH, symptomatic intracranial hemorrhage; SMT, standard medical treatment.

### Clinical outcomes of patients stratified by NIHSS score in EVT group (NIHSS score 0–9 vs. 10–25 vs. >25)

**Figure 2:**
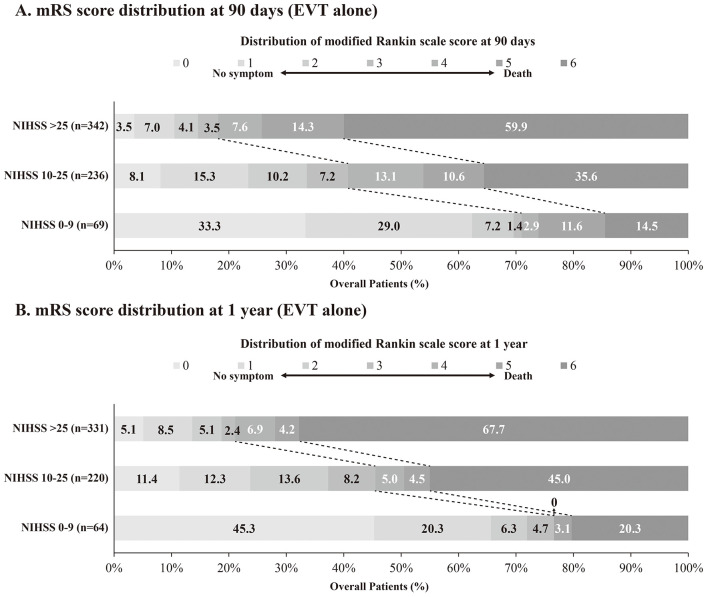
Distribution of modified Rankin Scale score at 90 days and 1 year in ABAO patients stratified by NIHSS score in EVT group (NIHSS score 0–9 vs. 10–25 vs. >25). The distributions of mRS scores at 90 days **(A)** and 1 year **(B)** in ABAO patients in EVT group are presented, stratified by NIHSS score. ABAO, acute basilar artery occlusion; EVT, endovascular treatment; mRS, modified Rankin scale; NIHSS, National Institutes of Health Stroke Scale; PSM, propensity score matching.

To evaluate the impact of baseline stroke severity on treatment outcomes, we stratified the 647 ABAO patients receiving EVT into three groups based on NIHSS scores: 0–9 (mild to moderate), 10–25 (moderate to severe), and >25 (extremely severe), and compared treatment effectiveness. Baseline characteristics of patients were shown in Table S5. Higher stroke severity was significantly associated with increased rates of AF (*P* = 0.008) and CHD (*P* = 0.014), lower baseline pc-ASPECTS (*P* < 0.001), and higher rates of general anesthesia (*P* < 0.001). Additionally, significant differences in ASITN/SIR grade distribution were observed across the three severity groups (*P* < 0.001). No significant differences were observed among the three groups for other baseline characteristics. Compared with the NIHSS 0–9 group, both NIHSS 10–25 (aOR 0.29, 95% CI 0.17–0.50, *P* < 0.001) and NIHSS > 25 (aOR 0.14, 95% CI 0.08–0.25, *P* < 0.001) were significantly associated with lower odds of achieving a favorable shift in mRS distribution (Figure 2A and Table S6). Similar trends were observed for outcomes of mRS 0–3, mRS 0–2, and mRS 0–1. Additionally, NIHSS 10–25 (aOR 2.42, 95% CI 1.06–5.49, *P* = 0.035) and NIHSS > 25 (aOR 5.16, 95% CI 2.30–11.61, *P* < 0.001) were significantly associated with higher odds of mortality compared with NIHSS 0–9. No significant differences in the odds of sICH within 48 h among the three severity groups. These findings persisted at 1-year follow-up, with similar long-term clinical outcomes observed between groups (Figure 2B and Table S7).

### Marginal effects and clinical outcomes

**Figure 3:**
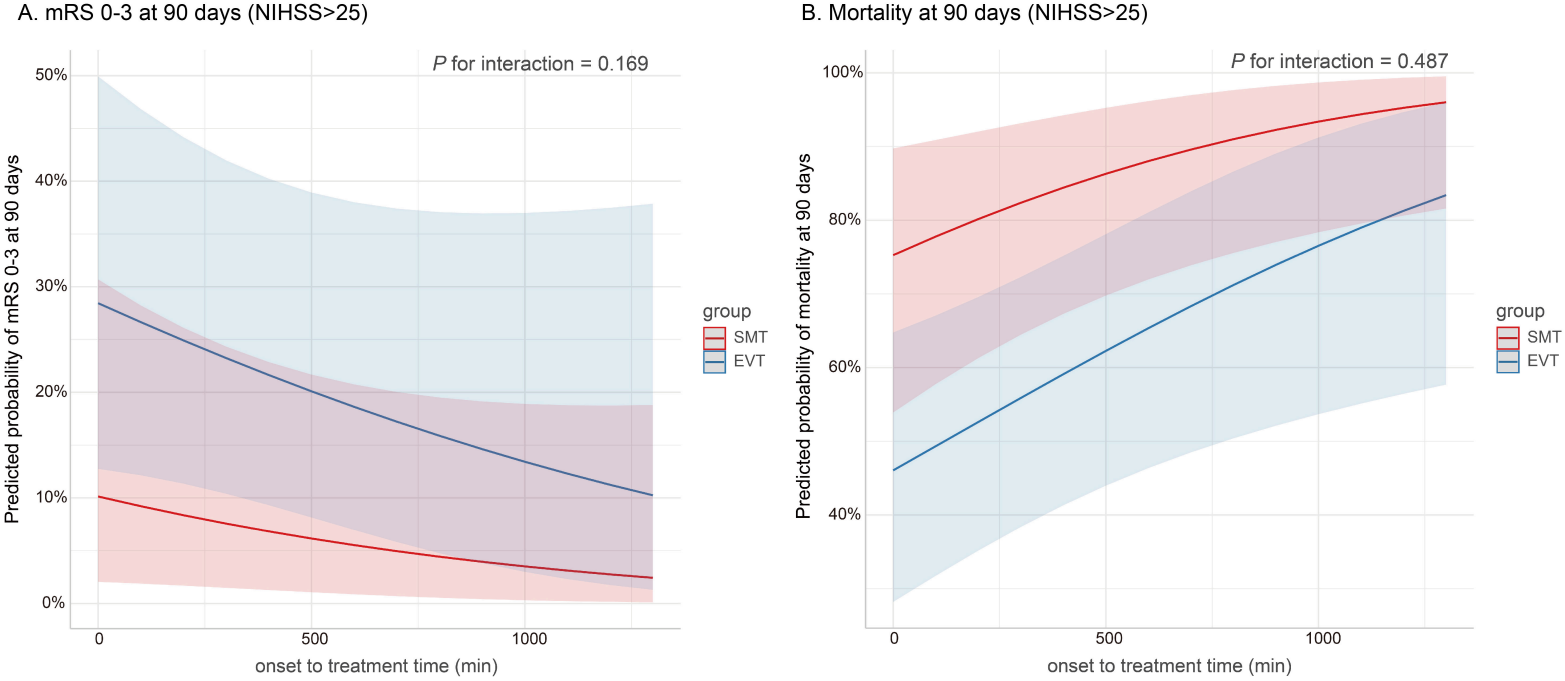
Association of onset to treatment time with the predicted probability of clinical outcomes in ABAO patients with extremely severe symptoms. The predicted probabilities of achieving mRS 0–3 and mortality by onset to treatment time among ABAO patients with extremely severe symptoms are presented in **(A)** and **(B)**. The predicted probability of achieving mRS 0–3 progressively decreases with longer onset to treatment time, while the predicted probability of mortality correspondingly increases. Compared to the SMT group, the EVT group exhibits higher predicted probabilities of mRS 0–3 and lower predicted probabilities of mortality. However, no interaction was found between the onset to treatment time and groups for either outcome (*P* for interaction = 0.169 and 0.487, respectively). Solid lines indicate predicted probabilities of outcomes; shaded areas represent 95% CIs. ABAO, acute basilar artery occlusion; CI, confidence interval; EVT, endovascular treatment; mRS, modified Rankin scale; SMT, standard medical treatment.

The predicted probabilities of achieving mRS 0–3 and mortality among ABAO patients with extremely severe symptoms (NIHSS score > 25) were estimated based on onset to treatment time, as illustrated in Figure 3. The predicted probability of achieving mRS 0–3 progressively decreased with increasing onset to treatment time, while the predicted probability of mortality correspondingly increased. The EVT group consistently demonstrated a higher predicted probability of achieving mRS 0–3 and a lower predicted probability of mortality compared with the SMT group. However, no interaction was observed between the onset to treatment time and groups for either outcome (*P* for interaction = 0.169 and 0.487, respectively).

The predicted probabilities of achieving mRS 0–3 and mortality among ABAO patients stratified by NIHSS score in the EVT group (NIHSS score 0–9 vs. 10–25 vs. >25) were estimated based on puncture to reperfusion time, as depicted in Figure S3. The predicted probability of achieving mRS 0–3 progressively decreased with longer puncture to reperfusion time, while the predicted probability of mortality correspondingly increased. Lower baseline stroke severity was associated with higher predicted probability of achieving mRS 0–3 and lower predicted probability of mortality. Additionally, no interaction was found between the puncture to reperfusion time and baseline stroke severity for either outcome (*P* for interaction = 0.322 and 0.869, respectively).

### Subgroup analyses

Subgroup analyses were conducted to explore potential heterogeneity in EVT treatment effects on mRS distribution across different patient populations. No significant heterogeneity was observed across all subgroups stratified by age, sex, baseline NIHSS score, baseline pc-ASPECTS, AF, ASITN/SIR grade, stroke etiology, occlusion site, IVT administration, and onset to treatment time (Figure S4).

## Discussion

This study investigated the effectiveness and safety of EVT in ABAO patients with extremely severe symptoms. Our findings demonstrated that EVT was significantly associated with improved functional outcomes in this patient population. Shorter onset to treatment time and puncture to reperfusion time were associated with higher predicted probabilities of achieving mRS 0–3 and lower predicted probabilities of mortality. Additionally, the benefit of EVT with longer onset-to-treatment intervals was negatively associated with increasing baseline stroke severity.

Patients with NIHSS score > 25 demonstrated a higher frequency of posterior circulation stroke and more commonly presented with impaired consciousness upon admission compared to patients with anterior stroke (12). The ATTENTION (5) and BAOCHE (6) trials provided compelling evidence for the efficacy and safety of EVT in patients with ABAO. Notably, these pivotal trials only analyzed patients with NIHSS score > 20 within subgroup analyses. A previous study demonstrated that EVT was safe and successful recanalization was strongly associated with better functional outcomes in patients with extremely severe anterior circulation ischemic stroke; however, the study lacked an SMT group for comparison (13). However, the real-world effectiveness and safety of EVT for ABAO patients with extremely severe symptoms remained unclear. Our findings demonstrated significant clinical benefits of EVT in ABAO patients with extremely severe symptoms at both short-term and long-term follow-up. These findings provide robust evidence to support clinical decision-making for EVT in this challenging patient population.

SICH is a serious complication after EVT for acute ischemic stroke, has been shown to be associated with poor functional prognosis and increased mortality (14, 15). The risk of sICH after IVT or EVT appears substantially lower in posterior circulation stroke patients compared to those with anterior circulation stroke (16, 17). Additionally, higher baseline NIHSS score has been identified as a predictor of sICH (18). In our cohort, although the EVT group had a significantly higher incidence of sICH compared with the SMT group among ABAO patients with extremely severe symptoms, the EVT group achieved superior clinical outcomes. Notably, we did not observe significant differences in sICH rates across varying baseline stroke severity groups among ABAO patients undergoing EVT. These observations suggest that EVT maintains an acceptable safety profile in this patient population.

The principle of “time is brain” emphasizes that treatment delays in acute ischemic stroke lead to irreversible neuronal loss of approximately 1.9 million neurons per minute (19). Previous studies have demonstrated that shorter onset to treatment time is significantly associated with better functional outcomes (20, 21). Consistent with previous findings, our results demonstrated that the predicted probability of achieving mRS 0–3 progressively declined with increasing onset to treatment time. These findings underscore the critical importance of reducing treatment delays to optimize functional outcomes in ABAO patients with extremely severe symptoms.

Shorter procedural time was associated with reduced risk of mortality and higher odds of favorable outcomes (22, 23). In patients with ABAO, the risk of complications and mortality increased by 0.5% and 1.5% with every 10-min increase in procedural time, respectively (23). Our findings also revealed that among ABAO patients treated with EVT, longer puncture to reperfusion time was associated with decreased predicted probability of achieving mRS 0–3 and increased predicted probability of mortality. Shortening EVT procedure time contributes to minimizing perioperative complications and reducing ischemic duration, thereby improving neurological outcomes (24). Therefore, maximizing technical proficiency and enhancing both the efficiency and quality of reperfusion are crucial for ABAO patients with extremely severe symptoms. Furthermore, when procedural time is significantly prolonged, clinicians should carefully assess the benefit-risk ratio of continuing the intervention to avoid futile or potentially harmful overtreatment.

### Limitations

However, this study has several limitations. First, the observational study design inherently carries the risk of selection bias, and our findings require validation through RCTs. Second, as the enrolled population was Chinese, the generalizability of our results to other ethnic populations requires further investigation. Finally, our findings necessitate confirmation in larger-scale studies.

## Conclusions

In ABAO patients with extremely severe symptoms, EVT was associated with superior functional outcomes and lower mortality compared to SMT alone. Minimizing onset to treatment time and puncture to reperfusion time is essential for optimizing clinical outcomes in this patient population. Additionally, the effectiveness and safety of EVT decreased progressively with increasing baseline stroke severity.

## Data Availability

The raw data supporting the conclusions of this article will be made available by the authors, without undue reservation.

## References

[1] Mattle HP Arnold M Lindsberg PJ Schonewille WJ Schroth G. Basilar artery occlusion. Lancet Neurol. (2011) 10:1002–14. doi: 10.1016/S1474-4422(11)70229-0 22014435

[2] Alemseged F Nguyen TN Alverne FM Liu X Schonewille WJ Nogueira RG. Endovascular therapy for basilar artery occlusion. Stroke. (2023) 54:1127–37. doi: 10.1161/STROKEAHA.122.040807 36722343

[3] Langezaal LCM van der Hoeven E Mont'Alverne FJA de Carvalho JJF Lima FO Dippel DWJ . Endovascular therapy for stroke due to basilar-artery occlusion. New Engl J Med. (2021) 384:1910–20. doi: 10.1056/NEJMoa2030297 34010530

[4] Liu X Dai Q Ye R Zi W Liu Y Wang H . Endovascular treatment versus standard medical treatment for vertebrobasilar artery occlusion (best): an open-label, randomised controlled trial. Lancet Neurol. (2020) 19:115–22. doi: 10.1016/S1474-4422(19)30395-3 31831388

[5] Tao C Nogueira RG Zhu Y Sun J Han H Yuan G . Trial of endovascular treatment of acute basilar-artery occlusion. New Engl J Med. (2022) 387:1361–72. doi: 10.1056/NEJMoa2206317 36239644

[6] Jovin TG Li C Wu L Wu C Chen J Jiang C . Trial of thrombectomy 6 to 24 hours after stroke due to basilar-artery occlusion. New Engl J Med. (2022) 387:1373–84. doi: 10.1056/NEJMoa2207576 36239645

[7] Zi W Qiu Z Wu D Li F Liu H Liu W . Assessment of endovascular treatment for acute basilar artery occlusion via a nationwide prospective registry. JAMA Neurol. (2020) 77:561–73. doi: 10.1001/jamaneurol.2020.0156 32080711 PMC7042866

[8] Guenego A Dargazanli C Weisenburger-Lile D Gory B Richard S Ducroux C . Thrombectomy for basilar artery occlusion with mild symptoms. World Neurosurg. (2021) 149:e400–14. doi: 10.1016/j.wneu.2021.02.010 33578025

[9] Wu D Guo F Liu D Hu R Shen Z Yang Y . Characteristics and prognosis of acute basilar artery occlusion in minor to moderate stroke and severe stroke after endovascular treatment: a multicenter retrospective study. Clin Neurol Neurosurg. (2021) 202:106504. doi: 10.1016/j.clineuro.2021.106504 33535127

[10] Kong W Yuan J Huang J Song J Zhao C Sang H . Outcomes of endovascular therapy in acute basilar artery occlusion with severe symptoms. JAMA Netw Open. (2021) 4:e2139550. doi: 10.1001/jamanetworkopen.2021.39550 34913974 PMC8678675

[11] von Kummer R Broderick JP Campbell BC Demchuk A Goyal M Hill MD . The heidelberg bleeding classification: classification of bleeding events after ischemic stroke and reperfusion therapy. Stroke. (2015). 46:2981–6. doi: 10.1161/STROKEAHA.115.010049 26330447

[12] Mazya MV Lees KR Collas D Rand VM Mikulik R Toni D . IV thrombolysis in very severe and severe ischemic stroke: results from the SITS-ISTR registry. Neurology. (2015) 85:2098–106. doi: 10.1212/WNL.0000000000002199 26546630 PMC4691682

[13] Bala F Bricout N Nouri N Cordonnier C Henon H Casolla B. Safety and outcomes of endovascular treatment in patients with very severe acute ischemic stroke. J Neurol. (2022) 269:2493–502. doi: 10.1007/s00415-021-10807-z 34618225

[14] Chen JH Su IC Lu YH Hsieh YC Chen CH Lin CJ . Predictive modeling of symptomatic intracranial hemorrhage following endovascular thrombectomy: insights from the nationwide treat-ais registry. J Stroke. (2025) 27:85–94. doi: 10.5853/jos.2024.04119 39916457 PMC11834349

[15] Mierzwa AT Nelson A Kasab SA Ortega Gutierrez S Vivanco-Suarez J Farooqui M . Predictors of outcome and symptomatic intracranial hemorrhage in acute basilar artery occlusions: analysis of the pc-search thrombectomy registry. Eur Stroke J. (2024) 9:583–91. doi: 10.1177/23969873241234713 38403924 PMC11418451

[16] Tong X Liao X Pan Y Cao Y Wang C Liu L . Intravenous thrombolysis is more safe and effective for posterior circulation stroke: data from the thrombolysis implementation and monitor of acute ischemic stroke in China (TIMS-China). Medicine (Baltimore). (2016) 95:e3848. doi: 10.1097/MD.0000000000003848 27310965 PMC4998451

[17] Weber R Minnerup J Nordmeyer H Eyding J Krogias C Hadisurya J . Thrombectomy in posterior circulation stroke: differences in procedures and outcome compared to anterior circulation stroke in the prospective multicentre revask registry. Eur J Neurol. (2019) 26:299–305. doi: 10.1111/ene.13809 30218610

[18] van der Ende NAM Kremers FCC van der Steen W Venema E Kappelhof M Majoie C . Symptomatic intracranial hemorrhage after endovascular stroke treatment: external validation of prediction models. Stroke. (2023) 54:476–87. doi: 10.1161/STROKEAHA.122.040065 36689584 PMC9855739

[19] Johnson LSM. “Time is brain:” DCDD-NRP invalidates the unified brain-based determination of death. Am J Bioeth. (2024) 24:84–86. doi: 10.1080/15265161.2024.2337436 38829595

[20] Jahan R Saver JL Schwamm LH Fonarow GC Liang L Matsouaka RA . Association between time to treatment with endovascular reperfusion therapy and outcomes in patients with acute ischemic stroke treated in clinical practice. JAMA. (2019) 322:252–63. doi: 10.1001/jama.2019.8286 31310296 PMC6635908

[21] Sang HF Yuan JJ Qiu ZM Zhang M Hu XG Liu WH . Association between time to endovascular therapy and outcomes in patients with acute basilar artery occlusion. Neurology. (2021) 97:e2152–63. doi: 10.1212/WNL.0000000000012858 34649885

[22] Sadeler A Finitsis S Olivot JM Richard S Marnat G Sibon I . Impact of time from symptom onset to puncture, and puncture to reperfusion, in endovascular therapy in the late time window (>6 h). Int J Stroke. (2025) 20:357–66. doi: 10.1177/17474930241300073 39501524

[23] Guo C Song J Li L Yang J Huang J Xie D . Association of procedure time with clinical and procedural outcome in patients with basilar occlusion undergoing embolectomy. Neurology. (2023) 101:e253–66. doi: 10.1212/WNL.0000000000207395 37202165 PMC10382271

[24] Su J Hu X Chen L Li R Tao C Yin Y . Predictors of good outcomes and mortality after thrombectomy for basilar artery occlusion within 12 hours of onset. J Neurointerv Surg. (2024) 17:e139–45. doi: 10.1136/jnis-2023-021057 38228387

