# Supplementary material for: Clinical outcomes of endovascular treatment for acute basilar artery occlusion patients with extremely severe symptoms
Source: Front Neurol. 2026 Jan 7;16:1736679. doi: 10.3389/fneur.2025.1736679 (PMC12819325; doi:10.3389/fneur.2025.1736679)
Supplement: Supplementary file 1 [file Supplementary_file_1.docx]

**Supplementary materials**

**Method S1. Propensity score matching for patients with extremely severe symptoms** **(NIHSS score >25).**

**Method S2. Propensity score matching for patients with moderate to severe symptoms (NIHSS score 10-25).**

**Figure S1. Flow chart of the study.**

**Figure S2. Distribution of modified Rankin scale score at 1 year in ABAO patients with extremely severe symptoms (NIHSS score >25).**

**Figure S3. Association of puncture to reperfusion time with the predicted probability of clinical outcomes in patients receiving EVT.**

**Figure S4. Subgroup analyses.**

**Table S1. Clinical outcomes at 1 year for ABAO patients with extremely severe symptoms (NIHSS score >25).**

**Table S2. Comparison of baseline characteristics between SMT and EVT groups in ABAO patients with moderate to severe symptoms (NIHSS score 10-25).**

**Table S3. Clinical outcomes at 90 days between SMT and EVT groups in ABAO patients with moderate to severe symptoms (NIHSS score 10-25).**

**Table S4. Clinical outcomes at 1 year between SMT and EVT groups in ABAO patients with moderate to severe symptoms (NIHSS score 10-25).**

**Table S5. Comparison of baseline characteristics in patients stratified by NIHSS score in EVT group (NIHSS score 0-9 versus 10-25 versus >25).**

**Table S6. Clinical outcomes at 90 days of patients stratified by NIHSS score in EVT group (NIHSS score 0-9 versus 10-25 versus >25).**

**Table S7. Clinical outcomes at 1 year of patients stratified by NIHSS score in EVT group (NIHSS score 0-9 versus 10-25 versus >25).**

**Method S1. Propensity score matching for patients with extremely severe symptoms (NIHSS score >25).**

(A) Distributions of propensity scores before and after propensity score matching for the treatment (EVT) and control group (SMT). (B) Scatter plots showing the respective propensity scores before and after propensity score matching for the treatment and control group. (C) Standardized mean differences before propensity score matching (red) and after propensity score matching (blue) for all selected baseline covariates.


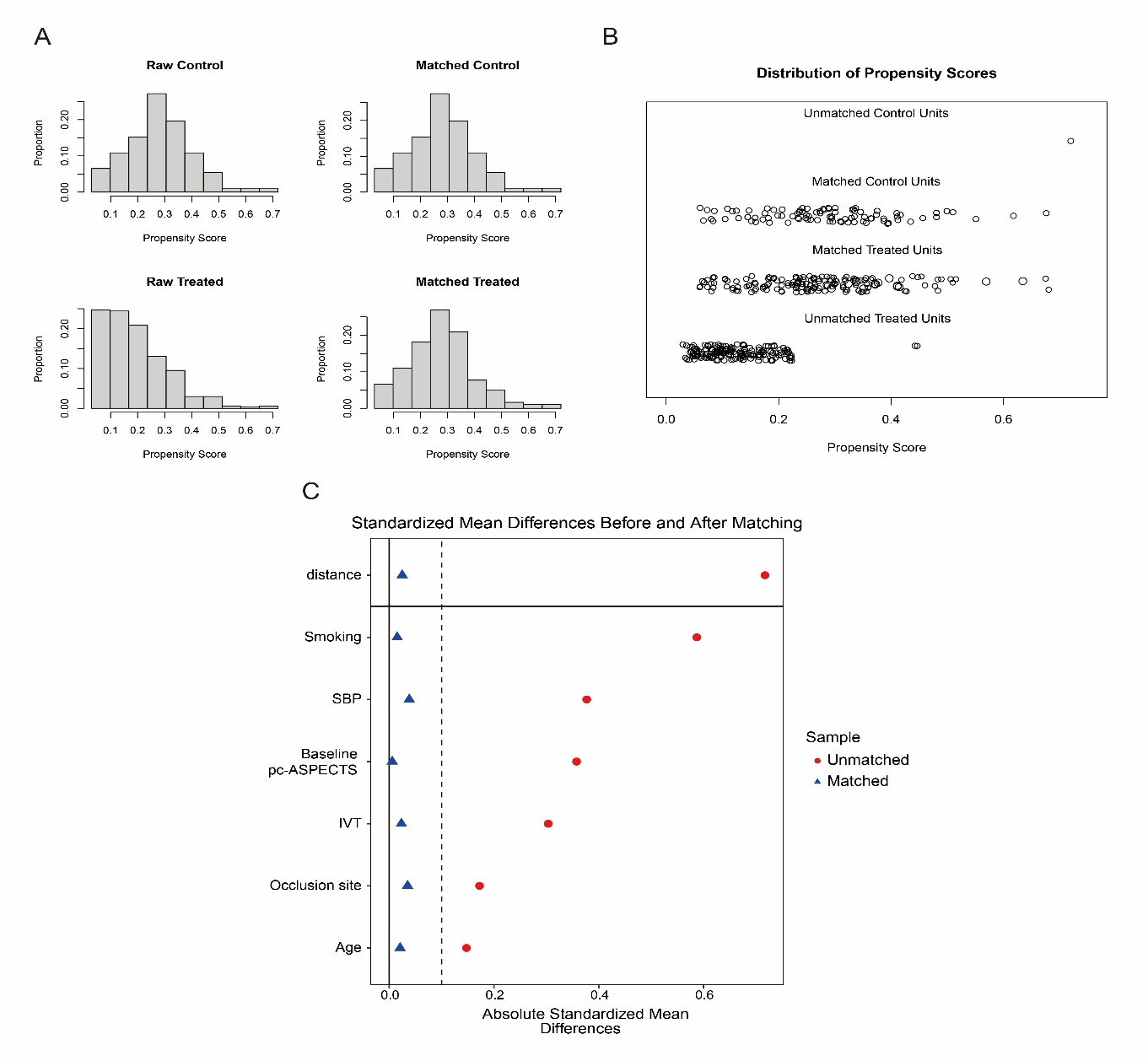
Abbreviations: EVT, endovascular treatment; IVT, intravenous thrombolysis; NIHSS, National Institutes of Health Stroke Scale; pc-ASPECTS, posterior circulation-Alberta Stroke Program Early Computed Tomography Score; SBP, systolic blood pressure; SMT, standard medical treatment.

**Method S2. Propensity score matching for patients with moderate to severe symptoms (NIHSS score 10-25).**

(A) Distributions of propensity scores before and after propensity score matching for the treatment (EVT) and control group (SMT). (B) Scatter plots showing the respective propensity scores before and after propensity score matching for the treatment and control group. (C) Standardized mean differences before propensity score matching (red) and after propensity score matching (blue) for all selected baseline covariates.


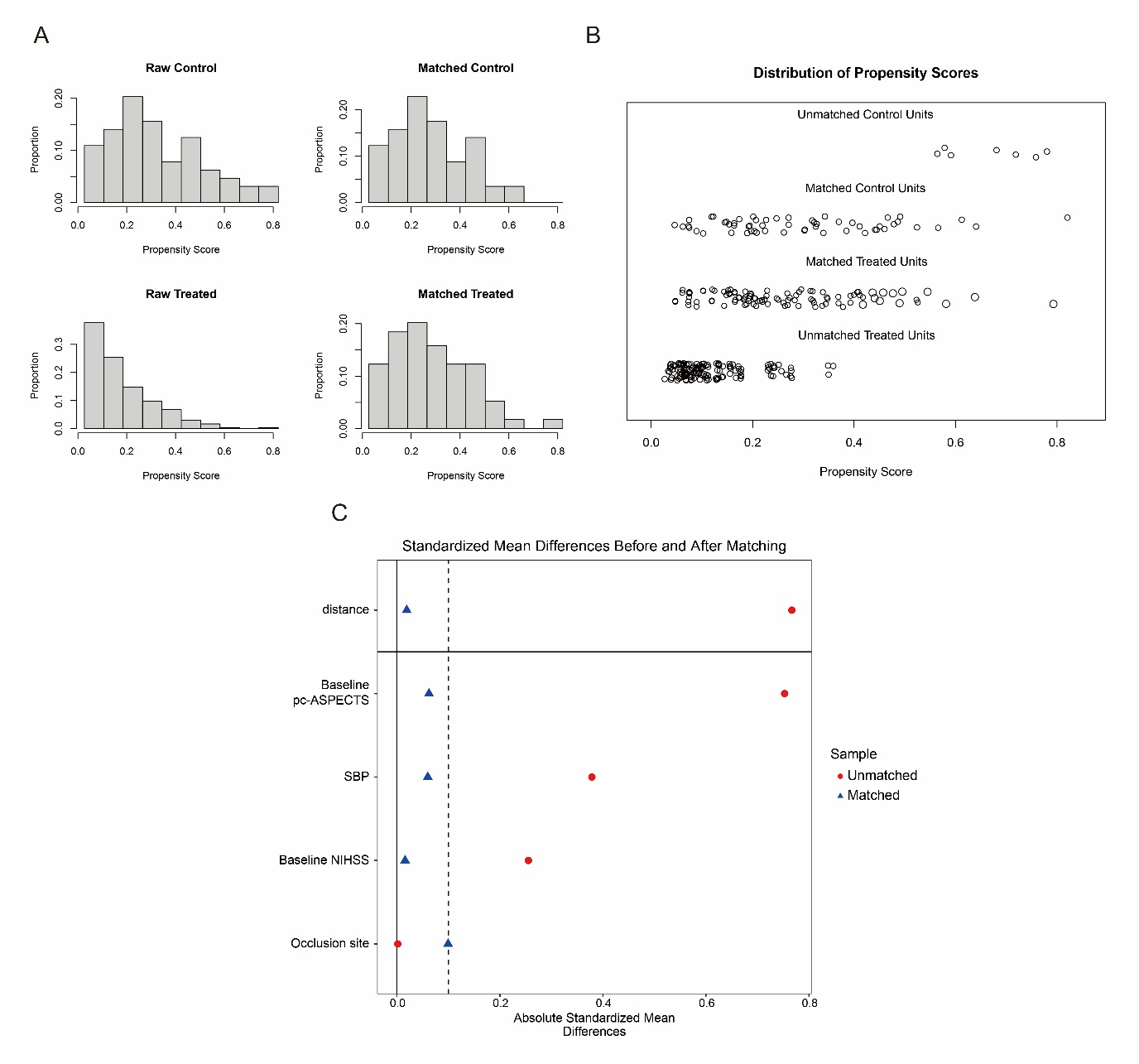
Abbreviations: EVT, endovascular treatment; NIHSS, National Institutes of Health Stroke Scale; pc-ASPECTS, posterior circulation-Alberta Stroke Program Early Computed Tomography Score; SBP, systolic blood pressure; SMT, standard medical treatment.

**Figure S1. Flow chart of the study.**

This figure shows the enrollment information of patients in this study.

Abbreviations: EVT, endovascular treatment; NIHSS, National Institutes of Health Stroke Scale; SMT, standard medical treatment.


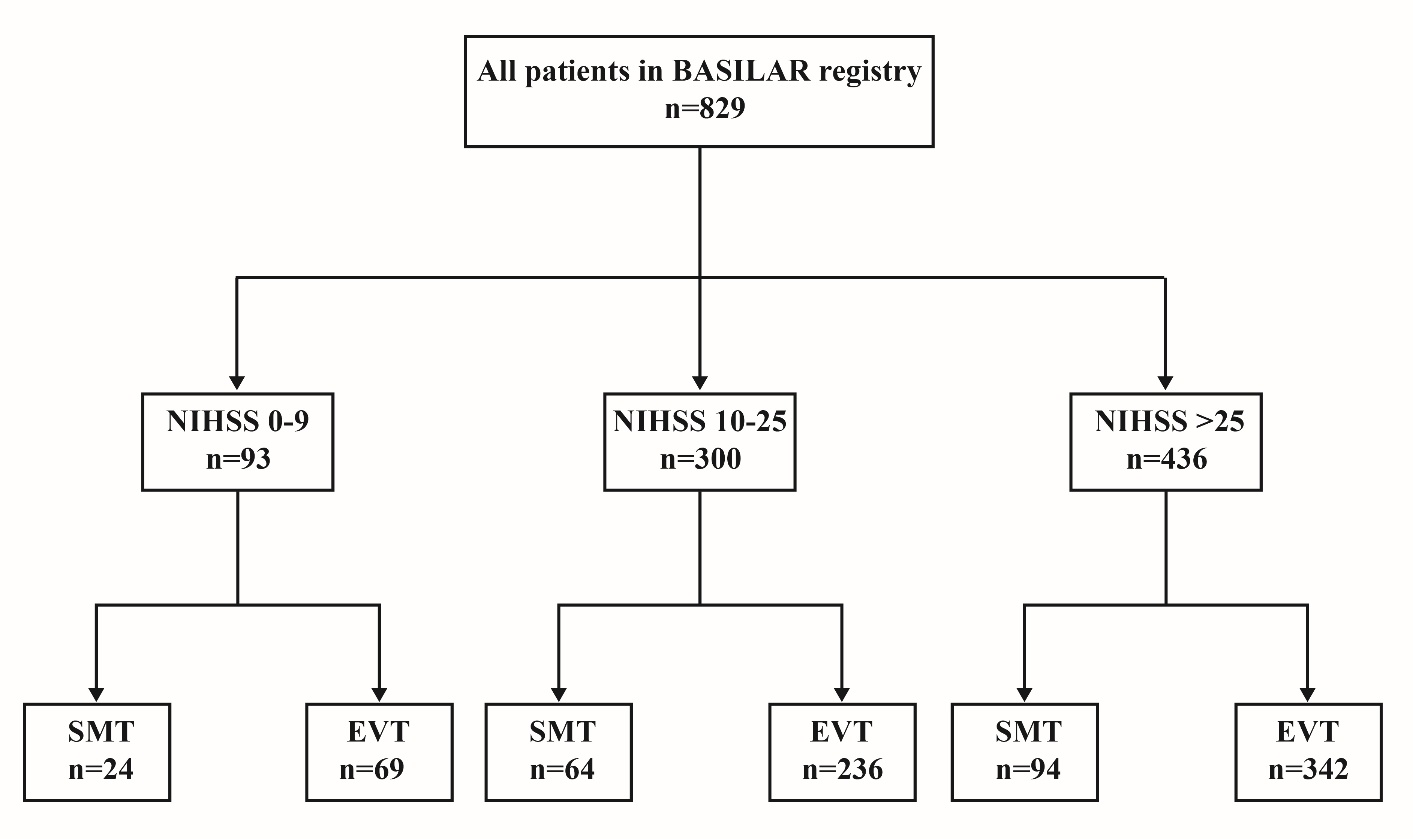


**Figure S2. Distribution of modified Rankin scale score at 1 year in ABAO patients with extremely severe symptoms (NIHSS score >25).**

The distributions of mRS scores at 1 year in ABAO patients with extremely severe symptoms are presented for the SMT and EVT groups before (A) and after (B) PSM.

Abbreviations: ABAO, acute basilar artery occlusion; EVT, endovascular treatment; mRS, modified Rankin Scale; PSM, propensity score matching; SMT, standard medical treatment.

**
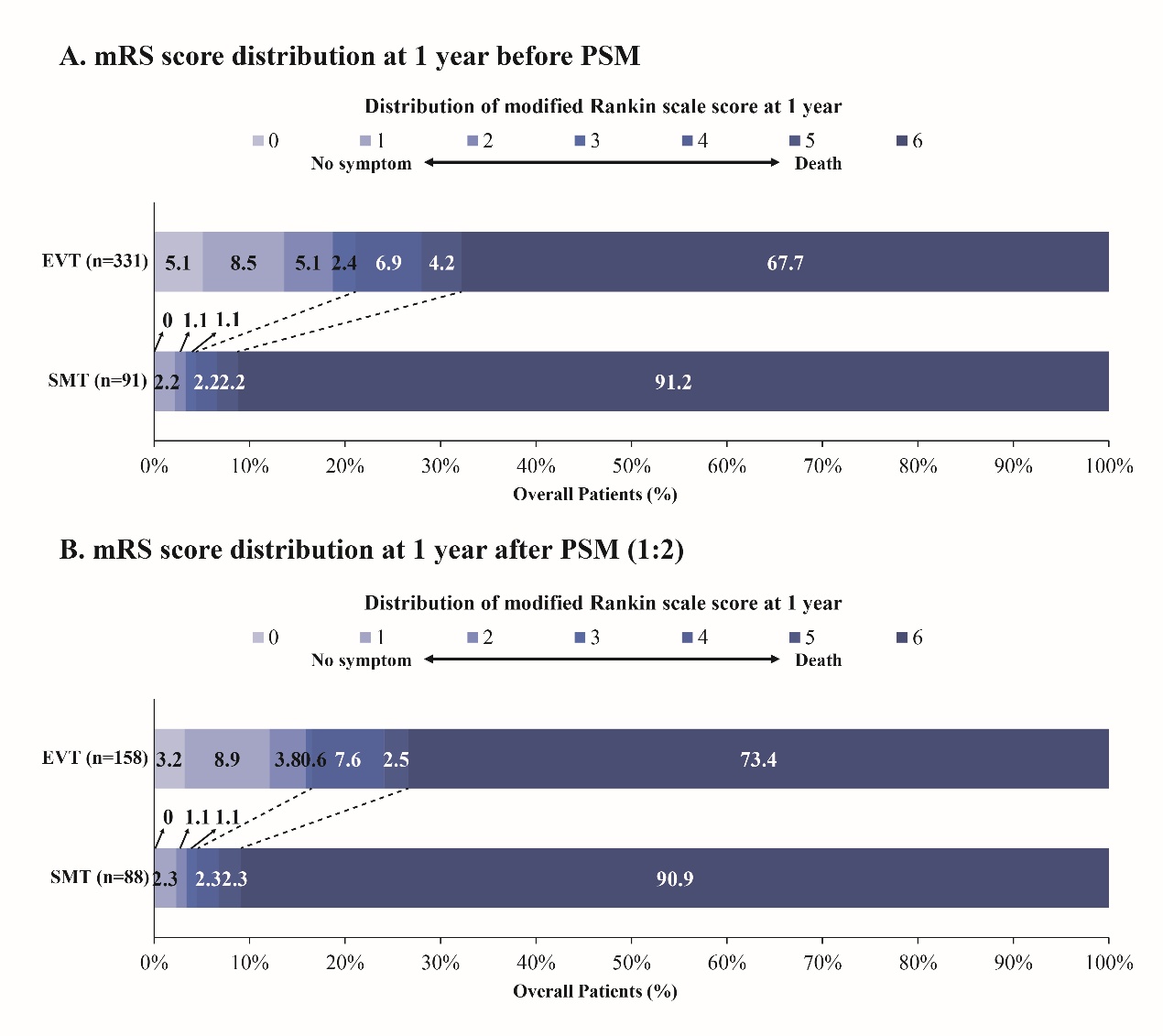
**

**Figure S3. Association of puncture to reperfusion time with the predicted probability of clinical outcomes in patients receiving EVT.**

The predicted probabilities of achieving mRS 0-3 and mortality by puncture to reperfusion time among patients with ABAO receiving EVT are presented in A and B. The predicted probability of achieving mRS 0-3 progressively decreased with longer puncture to reperfusion time, while the predicted probability of mortality increased correspondingly. Lower baseline stroke severity was associated with higher predicted probability of achieving mRS 0-3 and lower predicted probability of mortality. Additionally, no interaction was found between the puncture to reperfusion time and baseline stroke severity for either outcome (*P* for interaction = 0.322 and 0.869, respectively). Solid lines indicate predicted probabilities of outcomes; shaded areas represent 95% CIs.


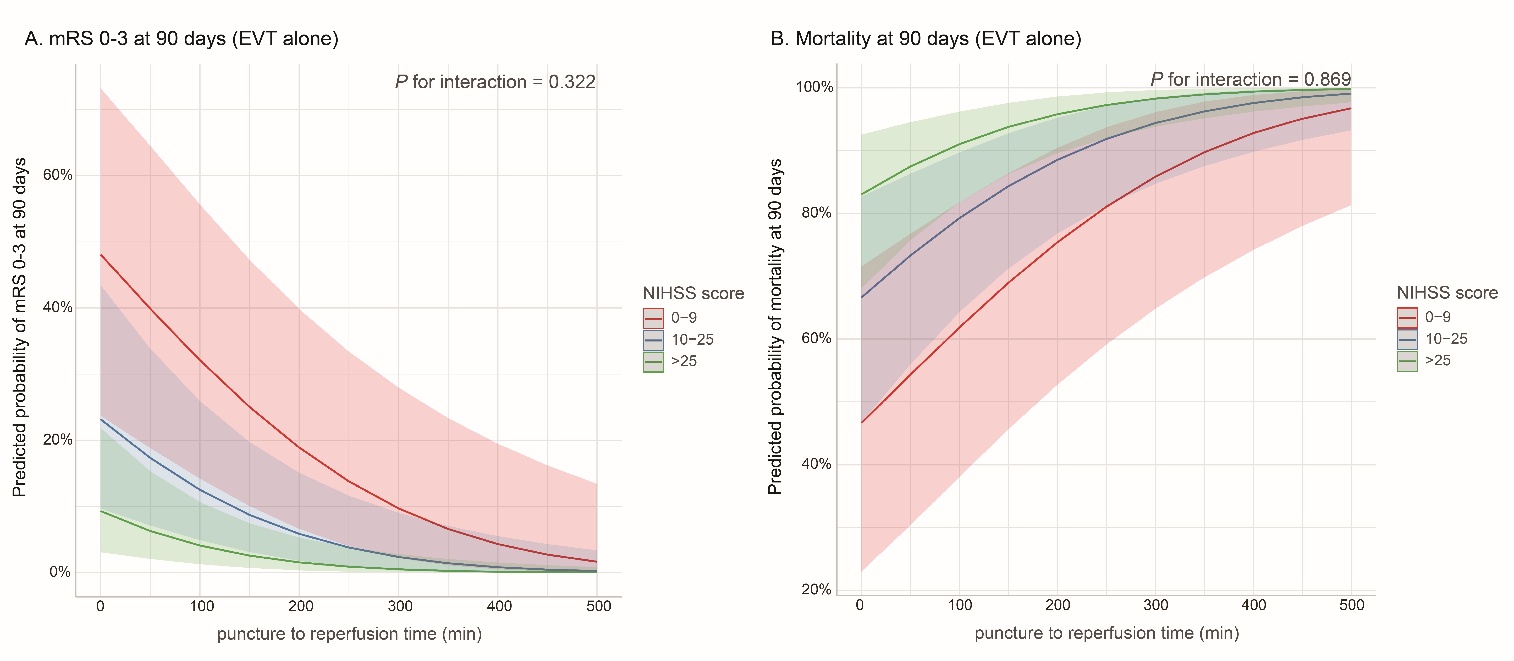
Abbreviations: ABAO, acute basilar artery occlusion; CI, confidence interval; EVT, endovascular treatment; mRS, modified Rankin Scale.

**Figure S4.** **Subgroup analyses.**

The forest plot illustrates the differences in odds ratios for mRS distribution at 90 days across subgroups of ABAO patients with extremely severe symptoms. Adjusted variables: age, sex, SBP, baseline NIHSS, baseline pc-ASPECTS, smoking history, ASITN/SIR grade, stroke etiology, occlusion site, and IVT.


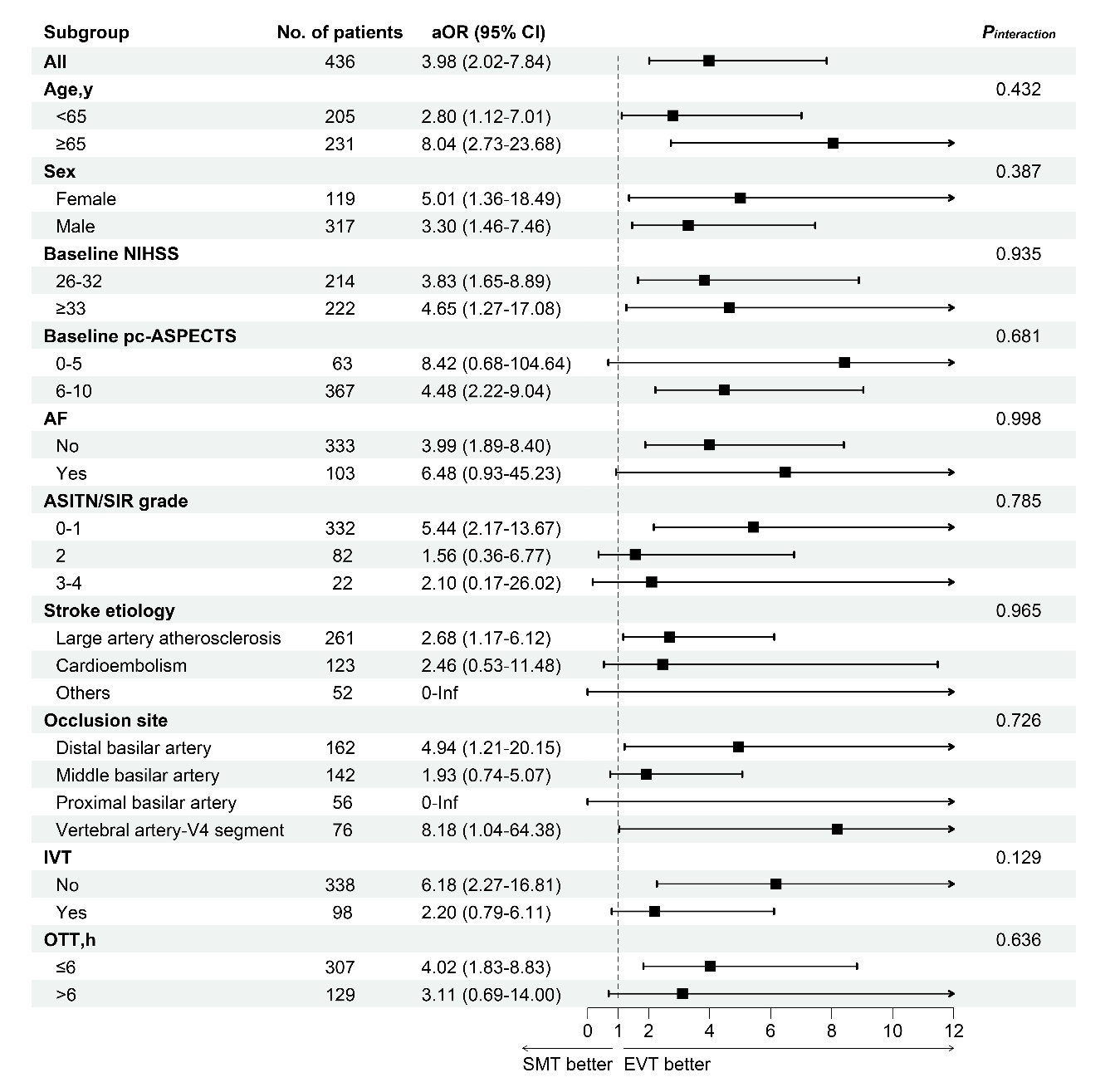
Abbreviations: ABAO, acute basilar artery occlusion; AF, atrial fibrillation; aOR, adjusted odds ratio; ASITN/SIR, American Society of Interventional and Therapeutic Neuroradiology/Society of Interventional Radiology; CI, confidence interval; EVT, endovascular treatment; IVT, intravenous thrombolysis; mRS, modified Rankin Scale; NIHSS, National Institutes of Health Stroke Scale; pc-ASPECTS, posterior circulation-Alberta Stroke Program Early Computed Tomography Score; OTT, onset to treatment time; SBP, systolic blood pressure; SMT, standard medical treatment.

Table S1. Clinical outcomes at 1 year for ABAO patients with extremely severe symptoms (NIHSS score >25).

| **Clinical outcomes** | **Before PSM** | | | | **After PSM (1:2)** | | | |
| --- | --- | --- | --- | --- | --- | --- | --- | --- |
|  | **SMT**  **(n=91), n (%)** | **EVT**  **(n=331), n (%)** | **Adjusted OR (95% CI)^A^** | ***P* value** | **SMT**  **(n=88), n (%)** | **EVT**  **(n=158), n (%)** | **Adjusted OR (95% CI)^A^** | ***P* value** |
| mRS score at 1 year^B^, median (IQR) | 6 (6-6) | 6 (4-6) | 5.31 (2.25-12.55) | <0.001 | 6 (6-6) | 6 (5-6) | 4.59 (1.86-11.34) | <0.001 |
| mRS 0-3 at 1 year ^C^ | 4 (4.4) | 70 (21.1) | 7.32 (2.14-25.07) | 0.002 | 4 (4.5) | 26 (16.5) | 5.76 (1.60-20.70) | 0.007 |
| mRS 0-2 at 1 year ^C^ | 3 (3.3) | 62 (18.7) | 7.60 (2.02-28.58) | 0.003 | 3 (3.4) | 25 (15.8) | 6.71 (1.70-26.48) | 0.007 |
| mRS 0-1 at 1 year ^C^ | 2 (2.2) | 45 (13.6) | 6.29 (1.34-29.47) | 0.020 | 2 (2.3) | 19 (12.0) | 6.98 (1.41-34.66) | 0.017 |
| Mortality at 1 year ^C^ | 83 (91.2) | 224 (67.7) | 0.18 (0.07-0.45) | <0.001 | 80 (90.9) | 116 (73.4) | 0.24 (0.09-0.59) | 0.002 |

^A^ adjusted for age, sex, SBP, baseline NIHSS, baseline pc-ASPECTS, smoking history, ASITN/SIR grade, stroke etiology, occlusion site, and IVT.

^B^ The common odds ratio was estimated from an ordinal logistic regression model.

^C^ The odds ratios were estimated from a binary logistic regression model.

Abbreviations: ASITN/SIR, American Society of Interventional and Therapeutic Neuroradiology/Society of Interventional Radiology; CI, confidence interval; EVT, endovascular treatment; IQR, interquartile range; IVT, intravenous thrombolysis; mRS, modified Rankin Scale; NIHSS, National Institutes of Health Stroke Scale; OR, odds ratio; pc-ASPECTS, posterior circulation-Alberta Stroke Program Early CT Score; PSM, propensity score matching; SBP, systolic blood pressure; SMT, standard medical treatment.

Table S2. Comparison of baseline characteristics between SMT and EVT groups in ABAO patients with moderate to severe symptoms (NIHSS score 10-25).

| **Characteristics** | **Before PSM** | | | | | | | **After PSM (1:2)** | | | |
| --- | --- | --- | --- | --- | --- | --- | --- | --- | --- | --- | --- |
|  | **All patients**  **(n=300)** | **SMT**  **(n=64)** | | | **EVT**  **(n=236)** | ***P***  **value** | | **All patients**  **(n=159)** | **SMT**  **(n=57)** | **EVT**  **(n=102)** | ***P***  **value** |
| Age, y, median (IQR) | 64 (57-74) | | 67 (60-76) | | 64 (56-73) | | 0.040 | 65 (58-74) | 67 (60-76) | 63 (57-73) | 0.080 |
| Sex, male, n (%) | 224 (74.7) | | 48 (75.0) | | 176 (74.6) | | 0.945 | 114 (71.7) | 42 (73.7) | 72 (70.6) | 0.678 |
| Medical history, n (%) |  | |  | |  | |  |  |  |  |  |
| Hypertension | 221 (73.7) | | 51 (79.7) | | 170 (72.0) | | 0.218 | 123 (77.4) | 45 (78.9) | 78 (76.5) | 0.720 |
| Diabetes mellitus | 72 (24.0) | | 14 (21.9) | | 58 (24.6) | | 0.654 | 38 (23.9) | 12 (21.1) | 26 (25.5) | 0.529 |
| Hyperlipidemia | 110 (36.7) | | 26 (40.6) | | 84 (35.6) | | 0.459 | 63 (39.6) | 23 (40.4) | 40 (39.2) | 0.888 |
| Smoking | 101 (33.7) | | 21 (32.8) | | 80 (33.9) | | 0.870 | 52 (32.7) | 18 (31.6) | 34 (33.3) | 0.821 |
| Ischemic stroke | 71 (23.7) | | 18 (28.1) | | 53 (22.5) | | 0.344 | 39 (24.5) | 17 (29.8) | 22 (21.6) | 0.246 |
| AF | 44 (14.7) | | 7 (10.9) | | 37 (15.7) | | 0.342 | 21 (13.2) | 6 (10.5) | 15 (14.7) | 0.455 |
| CHD | 40 (13.3) | | 11 (17.2) | | 29 (12.3) | | 0.306 | 24 (15.1) | 10 (17.5) | 14 (13.7) | 0.519 |
| Prodromal transient ischemic stroke or minor stroke | 153 (51.0) | | 37 (57.8) | | 116 (49.2) | | 0.219 | 87 (54.7) | 33 (57.9) | 54 (52.9) | 0.547 |
| SBP, mmHg, median (IQR) | 152 (135-170) | | 160 (144-175) | | 150 (134-168) | | 0.011 | 159 (140-177) | 156 (142-173) | 159 (138-182) | 0.947 |
| Baseline NIHSS score, median (IQR) | 18 (14-22) | | 17 (14-21) | | 18 (14-22) | | 0.087 | 17 (14-21) | 17 (14-22) | 18 (14-21) | 0.900 |
| Baseline pc-ASPECTS, median (IQR) | 8 (7-9) | | 7 (6-8) | | 8 (7-9) | | <0.001 | 7 (6-8) | 7 (6-8) | 7 (7-8) | 0.478 |
| ASITN/SIR grade, n (%) |  | |  | |  | |  |  |  |  |  |
| 0-1 | 143 (47.7) | | 35 (54.7) | | 108 (45.8) | | 0.435 | 83 (52.2) | 32 (56.1) | 51 (50.0) | 0.645 |
| 2 | 106 (35.3) | | 19 (29.7) | | 87 (36.9) | |  | 52 (32.7) | 16 (28.1) | 36 (35.3) |  |
| 3-4 | 51 (17.0) | | 10 (15.6) | | 41 (17.4) | |  | 24 (15.1) | 9 (15.8) | 15 (14.7) |  |
| Pre-stroke mRS score |  | |  | |  | |  |  |  |  |  |
| 0 | 249 (83.0) | | 52 (81.3) | | 197 (83.5) | | 0.245 | 131 (82.4) | 46 (80.7) | 85 (83.3) | 0.263 |
| 1 | 39 (13.0) | | 7 (10.9) | | 32 (13.6) | |  | 20 (12.6) | 6 (10.5) | 14 (13.7) |  |
| 2 | 12 (4.0) | | 5 (7.8) | | 7 (3.0) | |  | 8 (5.0) | 5 (8.8) | 3 (2.9) |  |
| Stroke etiology, n (%) |  | |  | |  | |  |  |  |  |  |
| LAA | 214 (71.3) | | 51 (79.7) | | 163 (69.1) | | 0.218 | 122 (76.7) | 47 (82.5) | 75 (73.5) | 0.418 |
| CE | 59 (19.7) | | 8 (12.5) | | 51 (21.6) | |  | 27 (17.0) | 7 (12.3) | 20 (19.6) |  |
| Others | 27 (9.0) | | 5 (7.8) | | 22 (9.3) | |  | 10 (6.3) | 3 (5.3) | 7 (6.9) |  |
| Occlusion site, n (%) |  | |  | |  | |  |  |  |  |  |
| Distal basilar artery | 77 (25.7) | | 8 (12.5) | | 69 (29.2) | | <0.001 | 33 (20.8) | 7 (12.3) | 26 (25.5) | 0.069 |
| Middle basilar artery | 116 (38.7) | | 40 (62.5) | | 76 (32.2) | |  | 78 (49.1) | 35 (61.4) | 43 (42.2) |  |
| Proximal basilar artery | 53 (17.7) | | 6 (9.4) | | 47 (19.9) | |  | 24 (15.1) | 6 (10.5) | 18 (17.6) |  |
| Vertebral artery-V4 segment | 54 (18.0) | | 10 (15.6) | | 44 (18.6) | |  | 24 (15.1) | 9 (15.8) | 15 (14.7) |  |
| IVT, n (%) | 57 (19.0) | | 11 (17.2) | | 46 (19.5) | | 0.677 | 28 (17.6) | 11 (19.3) | 17 (16.7) | 0.676 |
| Onset to imaging time, min, median (IQR) | 189 (85-362) | | 198 (103-388) | | 187 (79-334) | | 0.327 | 204 (94-379) | 182 (98-401) | 211 (93-364) | 0.933 |
| Onset to treatment time, min, median (IQR) | 232 (124-394) | | 242 (132-434) | | 232 (123-367) | | 0.397 | 242 (133-413) | 219 (127-453) | 248 (138-399) | 0.970 |
| Onset to puncture time, min, median (IQR)^a^ | NA | | NA | | 322 (217-488) | | NA | NA | NA | 330 (239-496) | NA |
| Puncture to reperfusion time, min, median (IQR)^b^ | NA | | NA | | 111 (74-159) | | NA | NA | NA | 120 (76-176) | NA |
| General anesthesia, n (%)^c^ | NA | NA | | 92 (39.7) | | | NA | NA | NA | 41 (41.0) | NA |
| mTICI score of 2b-3 at final angiogram, n (%) | 196 (65.3) | 3 (4.7) | | 193 (81.8) | | | <0.001 | 84 (52.8) | 3 (5.3) | 81 (79.4) | <0.001 |

^a^ Data were missing for 3 patients in the EVT group.

^b^ Data were missing for 3 patients in the EVT group.

^c^ Data were missing for 4 patients in the EVT group.

Abbreviation: AF, atrial fibrillation; ASITN/SIR, American Society of Interventional and Therapeutic Neuroradiology/Society of Interventional Radiology; CE, cardioembolism; CHD, coronary heart disease; CI, confidence interval; EVT, endovascular treatment; IQR, interquartile range; IVT, intravenous thrombolysis; LAA, large artery atherosclerosis; mRS, modified Rankin Scale; mTICI, modified Treatment in Cerebral Infarction; NA, not applicable; NIHSS, National Institutes of Health Stroke Scale; pc-ASPECTS, posterior circulation-Alberta Stroke Program Early Computed Tomography Score; PSM, propensity score matching; SBP, systolic blood pressure; SMT, standard medical treatment.

Table S3. Clinical outcomes at 90 days between SMT and EVT groups in ABAO patients with moderate to severe symptoms (NIHSS score 10-25).

| **Clinical outcomes** | **Before PSM** | | | | | **After PSM (1:2)** | | | |
| --- | --- | --- | --- | --- | --- | --- | --- | --- | --- |
|  | **SMT**  **(n=64), n (%)** | **EVT**  **(n=236), n (%)** | | **Adjusted OR (95% CI)^A^** | ***P* value** | **SMT**  **(n=57), n (%)** | **EVT**  **(n=102), n (%)** | **Adjusted OR (95% CI)^A^** | ***P* value** |
| mRS score at 90d^B^, median (IQR) | 6 (4-6) | | 4 (2-6) | 2.02 (1.09-3.77) | 0.026 | 6 (5-6) | 5 (3-6) | 2.52 (1.23-5.17) | 0.012 |
| mRS 0-3 at 90d^C^ | 6 (9.4) | | 96 (40.7) | 4.14 (1.46-11.79) | 0.008 | 6 (10.5) | 31 (30.4) | 3.92 (1.19-12.93) | 0.025 |
| mRS 0-2 at 90d^C^ | 5 (7.8) | | 79 (33.5) | 2.84 (0.95-8.48) | 0.062 | 5 (8.8) | 21 (20.6) | 1.99 (0.50-7.98) | 0.333 |
| mRS 0-1 at 90d^C^ | 4 (6.3) | | 55 (23.3) | 1.94 (0.58-6.52) | 0.283 | 4 (7.0) | 14 (13.7) | 1.37 (0.29-6.58) | 0.694 |
| Mortality at 90d^C^ | 39 (60.9) | | 84 (35.6) | 0.51 (0.25-1.02) | 0.056 | 37 (64.9) | 41 (40.2) | 0.35 (0.16-0.79) | 0.011 |
| sICH within 48h^a,C^ | 0 (0.0) | | 9 (3.8) | NA | NA | 0 (0.0) | 4 (4.0) | NA | NA |

^a^ Data were missing for 1 patient in the EVT group.

^A^ adjusted for age, sex, SBP, baseline NIHSS, baseline pc-ASPECTS, smoking history, ASITN/SIR grade, stroke etiology, occlusion site, and IVT.

^B^ The common odds ratio was estimated from an ordinal logistic regression model.

^C^ The odds ratios were estimated from a binary logistic regression model.

Abbreviations: ASITN/SIR, American Society of Interventional and Therapeutic Neuroradiology/Society of Interventional Radiology; CI, confidence interval; EVT, endovascular treatment; IQR, interquartile range; IVT, intravenous thrombolysis; mRS, modified Rankin Scale; NA, not applicable; NIHSS, National Institutes of Health Stroke Scale; OR, odds ratio; pc-ASPECTS, posterior circulation-Alberta Stroke Program Early CT Score; PSM, propensity score matching; SBP, systolic blood pressure; sICH, symptomatic intracranial hemorrhage; SMT, standard medical treatment.

Table S4. Clinical outcomes at 1 year between SMT and EVT groups in ABAO patients with moderate to severe symptoms (NIHSS score 10-25).

| **Clinical outcomes** | **Before PSM** | | | | **After PSM (1:2)** | | | |
| --- | --- | --- | --- | --- | --- | --- | --- | --- |
|  | **SMT**  **(n=56), n (%)** | **EVT**  **(n=220), n (%)** | **Adjusted OR (95% CI)^A^** | ***P* value** | **SMT**  **(n=50), n (%)** | **EVT**  **(n=95), n (%)** | **Adjusted OR (95% CI)^A^** | ***P* value** |
| mRS score at 1 year^B^, median (IQR) | 6 (6-6) | 4 (2-6) | 2.71 (1.19-6.18) | 0.017 | 6 (6-6) | 5 (2-6) | 2.84 (1.13-7.13) | 0.026 |
| mRS 0-3 at 1 year ^C^ | 9 (16.1) | 100 (45.5) | 2.77 (1.12-6.89) | 0.028 | 8 (16.0) | 36 (37.9) | 2.69 (0.96-7.53) | 0.060 |
| mRS 0-2 at 1 year ^C^ | 8 (14.3) | 82 (37.3) | 2.19 (0.82-5.89) | 0.120 | 7 (14.0) | 27 (28.4) | 2.01 (0.62-6.53) | 0.246 |
| mRS 0-1 at 1 year ^C^ | 7 (12.5) | 52 (23.6) | 1.00 (0.33-2.98) | 0.996 | 6 (12.0) | 13 (13.7) | 0.88 (0.20-3.99) | 0.871 |
| Mortality at 1 year ^C^ | 46 (82.1) | 99 (45.0) | 0.29 (0.12-0.70) | 0.006 | 41 (82.0) | 46 (48.4) | 0.23 (0.09-0.61) | 0.003 |

^A^ adjusted for age, sex, SBP, baseline NIHSS, baseline pc-ASPECTS, smoking history, ASITN/SIR grade, stroke etiology, occlusion site, and IVT.

^B^ The common odds ratio was estimated from an ordinal logistic regression model.

^C^ The odds ratios were estimated from a binary logistic regression model.

Abbreviations: ASITN/SIR, American Society of Interventional and Therapeutic Neuroradiology/Society of Interventional Radiology; CI, confidence interval; EVT, endovascular treatment; IQR, interquartile range; IVT, intravenous thrombolysis; mRS, modified Rankin Scale; NIHSS, National Institutes of Health Stroke Scale; OR, odds ratio; pc-ASPECTS, posterior circulation-Alberta Stroke Program Early CT Score; PSM, propensity score matching; SBP, systolic blood pressure; SMT, standard medical treatment.

Table S5. Comparison of baseline characteristics in patients stratified by NIHSS score in EVT group (NIHSS score 0-9 versus 10-25 versus >25).

| **Characteristics** | **All patients**  **(n=647)** | **NIHSS 0-9**  **(n=69)** | **NIHSS 10-25**  **(n=236)** | **NIHSS >25** | ***P***  **value** |
| --- | --- | --- | --- | --- | --- |
|  |  |  |  | **(n=342)** |  |
| Age, y, median (IQR) | 64 (56-73) | 64 (57-69) | 64 (56-73) | 65 (57-74) | 0.353 |
| Sex, male, n (%) | 483 (74.7) | 54 (78.3) | 176 (74.6) | 253 (74.0) | 0.757 |
| Medical history, n (%) |  |  |  |  |  |
| Hypertension | 451 (69.7) | 43 (62.3) | 170 (72.0) | 238 (69.6) | 0.303 |
| Diabetes mellitus | 149 (23.0) | 12 (17.4) | 58 (24.6) | 79 (23.1) | 0.459 |
| Hyperlipidemia | 214 (33.1) | 26 (37.7) | 84 (35.6) | 104 (30.4) | 0.296 |
| Smoking | 235 (36.3) | 33 (47.8) | 80 (33.9) | 122 (35.7) | 0.100 |
| Ischemic stroke | 140 (21.6) | 10 (14.5) | 53 (22.5) | 77 (22.5) | 0.313 |
| AF | 136 (21.0) | 11 (15.9) | 37 (15.7) | 88 (25.7) | 0.008 |
| CHD | 105 (16.2) | 7 (10.1) | 29 (12.3) | 69 (20.2) | 0.014 |
| Prodromal transient ischemic stroke or minor stroke | 302 (46.7) | 37 (53.6) | 116 (49.2) | 149 (43.6) | 0.197 |
| SBP, mmHg, median (IQR)^a^ | 150 (134-166) | 149 (134-163) | 150 (134-168) | 150 (132-166) | 0.725 |
| Baseline NIHSS score, median (IQR) | 27 (17-33) | 6 (5-8) | 18 (14-22) | 33 (30-35) | <0.001 |
| Baseline pc-ASPECTS, median (IQR)^b^ | 8 (7-9) | 8 (7-10) | 8 (7-9) | 8 (6-9) | <0.001 |
| ASITN/SIR grade, n (%) |  |  |  |  |  |
| 0-1 | 390 (60.3) | 22 (31.9) | 108 (45.8) | 260 (76.0) | <0.001 |
| 2 | 175 (27.0) | 20 (29.0) | 87 (36.9) | 68 (19.9) |  |
| 3-4 | 82 (12.7) | 27 (39.1) | 41 (17.4) | 14 (4.1) |  |
| Pre-stroke mRS score |  |  |  |  |  |
| 0 | 546 (84.4) | 61 (88.4) | 197 (83.5) | 288 (84.2) | 0.242 |
| 1 | 71 (11.0) | 5 (7.2) | 32 (13.6) | 34 (9.9) |  |
| 2 | 30 (4.6) | 3 (4.3) | 7 (3.0) | 20 (5.8) |  |
| Stroke etiology, n (%) |  |  |  |  |  |
| LAA | 418 (64.6) | 49 (71.0) | 163 (69.1) | 206 (60.2) | 0.056 |
| CE | 173 (26.7) | 18 (26.1) | 51 (21.6) | 104 (30.4) |  |
| Others | 56 (8.7) | 2 (2.9) | 22 (9.3) | 32 (9.4) |  |
| Occlusion site, n (%) |  |  |  |  |  |
| Distal basilar artery | 222 (34.3) | 22 (31.9) | 69 (29.2) | 131 (38.3) | 0.311 |
| Middle basilar artery | 195 (30.1) | 22 (31.9) | 76 (32.2) | 97 (28.4) |  |
| Proximal basilar artery | 107 (16.5) | 10 (14.5) | 47 (19.9) | 50 (14.6) |  |
| Vertebral artery-V4 segment | 123 (19.0) | 15 (21.7) | 44 (18.6) | 64 (18.7) |  |
| IVT, n (%) | 119 (18.4) | 7 (10.1) | 46 (19.5) | 66 (19.3) | 0.173 |
| Onset to imaging time, min, median (IQR) | 210 (88-355) | 244 (41-386) | 187 (79-334) | 214 (102-352) | 0.356 |
| Onset to treatment time, min, median (IQR) | 246 (132-390) | 293 (89-420) | 232 (123-367) | 248 (141-394) | 0.438 |
| Onset to puncture time, min, median (IQR)^c^ | 328 (220-493) | 351 (265-618) | 322 (217-488) | 320 (220-487) | 0.222 |
| Puncture to reperfusion time, min, median (IQR)^d^ | 105 (71-151) | 103 (66-166) | 111 (74-159) | 102 (70-142) | 0.233 |
| General anesthesia, n (%)^e^ | 257 (40.2) | 13 (18.8) | 92 (39.7) | 152 (45.0) | <0.001 |
| mTICI score of 2b-3 at final angiogram, n (%) | 522 (80.7) | 62 (89.9) | 193 (81.8) | 267 (78.1) | 0.067 |

^a^ Data were missing for 1 patient in the NIHSS 0-9 group and 2 patients in the NIHSS >25 group.

^b^ Data were missing for 4 patients in the NIHSS >25 group.

^c^ Data were missing for 3 patients in the NIHSS 10-25 group.

^d^ Data were missing for 3 patients in the NIHSS 10-25 group.

^e^ Data were missing for 4 patients in the NIHSS 10-25 group and 4 patients in the NIHSS >25 group.

Abbreviation: AF, atrial fibrillation; ASITN/SIR, American Society of Interventional and Therapeutic Neuroradiology/Society of Interventional Radiology; CE, cardioembolism; CHD, coronary heart disease; CI, confidence interval; EVT, endovascular treatment; IQR, interquartile range; IVT, intravenous thrombolysis; LAA, large artery atherosclerosis; mRS, modified Rankin Scale; mTICI, modified Treatment in Cerebral Infarction; NIHSS, National Institutes of Health Stroke Scale; pc-ASPECTS, posterior circulation-Alberta Stroke Program Early Computed Tomography Score; PSM, propensity score matching; SBP, systolic blood pressure; SMT, standard medical treatment.

Table S6. Clinical outcomes at 90 days of patients stratified by NIHSS score in EVT group (NIHSS score 0-9 versus 10-25 versus >25).

| **Clinical outcomes** | **Groups** | **Frequencies** | **Unadjusted OR (95% CI)** | ***P* value** | **Adjusted OR (95% CI)^A^** | ***P* value** |  |
| --- | --- | --- | --- | --- | --- | --- | --- |
| mRS score at 90d^B^, median (IQR) | | NIHSS 0-9 | 1 (0-5) | reference |  | reference |  |
|  | | NIHSS 10-25 | 4 (2-6) | 0.22 (0.13-0.37) | <0.001 | 0.29 (0.17-0.50) | <0.001 |
|  | | NIHSS >25 | 6 (4-6) | 0.08 (0.05-0.13) | <0.001 | 0.14 (0.08-0.25) | <0.001 |
| mRS 0-3 at 90d^C^ | NIHSS 0-9 | 49 (71.0) | reference |  | reference |  |  |
|  | NIHSS 10-25 | 96 (40.7) | 0.28 (0.16-0.50) | <0.001 | 0.32 (0.16-0.63) | <0.001 |  |
|  | NIHSS >25 | 62 (18.1) | 0.09 (0.05-0.16) | <0.001 | 0.13 (0.07-0.27) | <0.001 |  |
| mRS 0-2 at 90d^C^ | NIHSS 0-9 | 48 (69.6) | reference |  | reference |  |  |
|  | NIHSS 10-25 | 79 (33.5) | 0.22 (0.12-0.39) | <0.001 | 0.22 (0.11-0.43) | <0.001 |  |
|  | NIHSS >25 | 50 (14.6) | 0.08 (0.04-0.14) | <0.001 | 0.10 (0.05-0.20) | <0.001 |  |
| mRS 0-1 at 90d^C^ | NIHSS 0-9 | 43 (62.3) | reference |  | reference |  |  |
|  | NIHSS 10-25 | 55 (23.3) | 0.18 (0.10-0.33) | <0.001 | 0.17 (0.09-0.34) | <0.001 |  |
|  | NIHSS >25 | 36 (10.5) | 0.07 (0.04-0.13) | <0.001 | 0.11 (0.05-0.22) | <0.001 |  |
| Mortality at 90d^C^ | NIHSS 0-9 | 10 (14.5) | reference |  | reference |  |  |
|  | NIHSS 10-25 | 84 (35.6) | 3.26 (1.59-6.71) | 0.001 | 2.42 (1.06-5.49) | 0.035 |  |
|  | NIHSS >25 | 205 (59.9) | 8.83 (4.37-17.86) | <0.001 | 5.16 (2.30-11.61) | <0.001 |  |
| sICH within 48h^a,C^ | NIHSS 0-9 | 1 (1.4) | reference |  | reference |  |  |
|  | NIHSS 10-25 | 9 (3.8) | 2.71 (0.34-21.76) | 0.349 | 1.71 (0.20-14.42) | 0.623 |  |
|  | NIHSS >25 | 35 (10.5) | 8.01 (1.08-59.52) | 0.042 | 4.75 (0.61-37.21) | 0.138 |  |

^a^ Data were missing for 1 patient in the NIHSS 10-25 group and 10 patients in the NIHSS >25 group.

^A^ adjusted for age, sex, baseline pc-ASPECTS, AF, CHD, ASITN/SIR grade, stroke etiology, anesthesia type, and reperfusion status.

^B^ The common odds ratio was estimated from an ordinal logistic regression model.

^C^ The odds ratios were estimated from a binary logistic regression model.

Abbreviations: AF, atrial fibrillation; ASITN/SIR, American Society of Interventional and Therapeutic Neuroradiology/Society of Interventional Radiology; CI, confidence interval; CHD, coronary heart disease; EVT, endovascular treatment; IQR, interquartile range; IVT, intravenous thrombolysis; mRS, modified Rankin Scale; NIHSS, National Institutes of Health Stroke Scale; OR, odds ratio; pc-ASPECTS, posterior circulation-Alberta Stroke Program Early CT Score; SBP, systolic blood pressure; sICH, symptomatic intracranial hemorrhage; SMT, standard medical treatment.

Table S7. Clinical outcomes at 1 year of patients stratified by NIHSS score in EVT group (NIHSS score 0-9 versus 10-25 versus >25).

| **Clinical outcomes** | **Groups** | **Frequencies** | **Unadjusted OR (95% CI)** | ***P* value** | **Adjusted OR (95% CI)^A^** | ***P* value** |  |
| --- | --- | --- | --- | --- | --- | --- | --- |
| mRS score at 1 year^B^, median (IQR) | | NIHSS 0-9 | 1 (0-3) | reference |  | reference |  |
|  | | NIHSS 10-25 | 4 (2-6) | 0.19 (0.11-0.33) | <0.001 | 0.24 (0.14-0.43) | <0.001 |
|  | | NIHSS >25 | 6 (4-6) | 0.08 (0.05-0.13) | <0.001 | 0.13 (0.07-0.24) | <0.001 |
| mRS 0-3 at 1 year^C^ | NIHSS 0-9 | 49 (76.6) | reference |  | reference |  |  |
|  | NIHSS 10-25 | 100 (45.5) | 0.26 (0.14-0.48) | <0.001 | 0.30 (0.14-0.63) | 0.001 |  |
|  | NIHSS >25 | 70 (21.1) | 0.08 (0.04-0.16) | <0.001 | 0.13 (0.06-0.28) | <0.001 |  |
| mRS 0-2 at 1 year^C^ | NIHSS 0-9 | 46 (71.9) | reference |  | reference |  |  |
|  | NIHSS 10-25 | 82 (37.3) | 0.23 (0.13-0.43) | <0.001 | 0.25 (0.12-0.50) | <0.001 |  |
|  | NIHSS >25 | 62 (18.7) | 0.09 (0.05-0.17) | <0.001 | 0.13 (0.07-0.28) | <0.001 |  |
| mRS 0-1 at 1 year^C^ | NIHSS 0-9 | 42 (65.6) | reference |  | reference |  |  |
|  | NIHSS 10-25 | 52 (23.6) | 0.16 (0.09-0.30) | <0.001 | 0.14 (0.07-0.29) | <0.001 |  |
|  | NIHSS >25 | 45 (13.6) | 0.08 (0.05-0.15) | <0.001 | 0.11 (0.05-0.22) | <0.001 |  |
| Mortality at 1 year^C^ | NIHSS 0-9 | 13 (20.3) | reference |  | reference |  |  |
|  | NIHSS 10-25 | 99 (45.0) | 3.21 (1.65-6.24) | <0.001 | 2.43 (1.13-5.22) | 0.023 |  |
|  | NIHSS >25 | 224 (67.7) | 8.21 (4.28-15.75) | <0.001 | 4.58 (2.15-9.77) | <0.001 |  |

^A^ adjusted for age, sex, baseline pc-ASPECTS, AF, CHD, ASITN/SIR grade, stroke etiology, anesthesia type, and reperfusion status.

^B^ The common odds ratio was estimated from an ordinal logistic regression model.

^C^ The odds ratios were estimated from a binary logistic regression model.

Abbreviations: AF, atrial fibrillation; ASITN/SIR, American Society of Interventional and Therapeutic Neuroradiology/Society of Interventional Radiology; CI, confidence interval; CHD, coronary heart disease; EVT, endovascular treatment; IQR, interquartile range; IVT, intravenous thrombolysis; mRS, modified Rankin Scale; NIHSS, National Institutes of Health Stroke Scale; OR, odds ratio; pc-ASPECTS, posterior circulation-Alberta Stroke Program Early CT Score; SBP, systolic blood pressure; sICH, symptomatic intracranial hemorrhage; SMT, standard medical treatment.
